# Plasma Gradient of Soluble Urokinase-Type Plasminogen Activator Receptor Is Linked to Pathogenic Plasma Proteome and Immune Transcriptome and Stratifies Outcomes in Severe COVID-19

**DOI:** 10.3389/fimmu.2021.738093

**Published:** 2021-10-28

**Authors:** Jafar Sarif, Deblina Raychaudhuri, Ranit D’Rozario, Purbita Bandopadhyay, Praveen Singh, Priyanka Mehta, Md. Asmaul Hoque, Bishnu Prasad Sinha, Manoj Kushwaha, Shweta Sahni, Priti Devi, Partha Chattopadhyay, Shekhar Ranjan Paul, Yogiraj Ray, Kausik Chaudhuri, Sayantan Banerjee, Debajyoti Majumdar, Bibhuti Saha, Biswanath Sharma Sarkar, Prasun Bhattacharya, Shilpak Chatterjee, Sandip Paul, Pramit Ghosh, Rajesh Pandey, Shantanu Sengupta, Dipyaman Ganguly

**Affiliations:** ^1^ Indian Institute of Chemical Biology (IICB)-Translational Research Unit of Excellence, Council of Scientific & Industrial Research (CSIR)-Indian Institute of Chemical Biology, Kolkata, India; ^2^ Department of Biological Sciences, Academy of Scientific and Innovative Research (AcSIR), Ghaziabad, India; ^3^ Cardiorespiratory Disease Biology, Council of Scientific & Industrial Research (CSIR)-Institute of Genomics and Integrative Biology, New Delhi, India; ^4^ INtegrative GENomics of HOst-PathogEn (INGEN-HOPE) Laboratory, Council of Scientific & Industrial Research (CSIR)-Institute of Genomics and Integrative Biology, New Delhi, India; ^5^ Department of Medicine, Infectious Diseases and Beliaghata General (ID & BG) Hospital, Kolkata, India; ^6^ Department of Tropical Medicine, School of Tropical Medicine, Kolkata, India; ^7^ Department of Immunohematology and Blood Transfusion, Medical College, Kolkata, India; ^8^ Department of Community Medicine, Deben Mahata Government Medical College & Hospital, Purulia, India

**Keywords:** COVID-19, soluble uPAR, Plaur, myeloid cells, prognosis, ARDS, proteomics

## Abstract

Disease caused by SARS-CoV-2 coronavirus (COVID-19) led to significant morbidity and mortality worldwide. A systemic hyper-inflammation characterizes severe COVID-19 disease, often associated with acute respiratory distress syndrome (ARDS). Blood biomarkers capable of risk stratification are of great importance in effective triage and critical care of severe COVID-19 patients. Flow cytometry and next-generation sequencing were done on peripheral blood cells and urokinase-type plasminogen activator receptor (suPAR), and cytokines were measured from and mass spectrometry-based proteomics was done on plasma samples from an Indian cohort of COVID-19 patients. Publicly available single-cell RNA sequencing data were analyzed for validation of primary data. Statistical analyses were performed to validate risk stratification. We report here higher plasma abundance of suPAR, expressed by an abnormally expanded myeloid cell population, in severe COVID-19 patients with ARDS. The plasma suPAR level was found to be linked to a characteristic plasma proteome, associated with coagulation disorders and complement activation. Receiver operator characteristic curve analysis to predict mortality identified a cutoff value of suPAR at 1,996.809 pg/ml (odds ratio: 2.9286, 95% confidence interval 1.0427–8.2257). Lower-than-cutoff suPAR levels were associated with a differential expression of the immune transcriptome as well as favorable clinical outcomes, in terms of both survival benefit (hazard ratio: 0.3615, 95% confidence interval 0.1433–0.912) and faster disease remission in our patient cohort. Thus, we identified suPAR as a key pathogenic circulating molecule linking systemic hyperinflammation to the hypercoagulable state and stratifying clinical outcomes in severe COVID-19 patients with ARDS.

## Introduction

The ongoing pandemic caused by the severe acute respiratory syndrome causing coronavirus 2 (SARS-COV-2) has resulted in close to 215 million documented infections and close to 4.5 million deaths. The respiratory disease caused by SARS-COV-2, or COVID-19, progresses to acute respiratory distress syndrome (ARDS), often with fatal outcomes, in some patients (1). Severe COVID-19 is characterized by systemic hyper-inflammation, the key manifestations being a systemic cytokine deluge and an abnormal myeloid expansion among circulating immune cells (2–5). In addition to the hyper-inflammation, patients with severe COVID-19 also present with intravascular coagulation as well as abnormal complement activation (6–9). Thus, the systemic cytokine surge, a hypercoagulable state, and tissue damage mediated by complement activation are the three established pathogenic triads in these patients. Risk-stratifying biomarkers, which can be probed early enough in severe COVID-19 patients, can be useful as pre-assessors for effective triage or intensive care in low-resource settings.

Single-cell RNA sequencing (scRNAseq) studies in severe COVID-19 revealed an abnormally expanded circulating myeloid cell compartment as well as an enriched expression of urokinase-type plasminogen activator receptor (PLAUR gene, expressing uPAR protein) (3, 4). UPAR (or CD87) is a glycosylphosphatidylinositol (GPI)-anchored receptor present on the surface of various cells, including immune cells, viz., monocytes, macrophages, and neutrophils. Cell-surface uPAR binds uPA and transforms plasminogen into plasmin, which in turn affects fibrinolysis and clot resolution as well as initiates a proteolytic cascade to degrade the components of the ECM (10–12). UPAR also lies within the complex regulatory network of the complement activation and complement-mediated pathogen or host cell clearance (11–13).

Increased plasma abundance of soluble uPAR has been documented widely in chronic inflammatory contexts (6); thus, it is also proposed to be a potential pathogenic molecule involved in the acute systemic hyper-inflammation in COVID-19 (10, 14). In a cohort of COVID-19 patients from India, originally recruited for a randomized control trial on convalescent plasma therapy, we found significantly high plasma levels of soluble uPAR in severe COVID-19 patients early in the course of severity, which correlated with an expanded myeloid cell compartment in circulation. A characteristic proteomics signature of activation of coagulation cascade as well as complement activation was found to be associated with higher plasma concentrations of suPAR, as were specific immune-related pathways enriched in peripheral blood transcriptome analysis. Finally, we found that patients suffering from ARDS in severe COVID-19 but with plasma levels of suPAR below a computed cutoff value registered significantly more favorable disease outcomes.

## Materials and Methods

### Patient Characteristics

COVID-19 patients with mild symptoms (N = 16) or ARDS (N = 77) were recruited at the ID & BG Hospital, Kolkata, India (detailed group-wise characteristics are depicted in **Supplemental Table 1**). Peripheral blood sampling in EDTA was done on the day of enrolment with due ethical approval from the institutional review boards of ID & BG Hospital, Kolkata, India (IDBGH/Ethics/2429), and CSIR-Indian Institute of Chemical Biology, Kolkata, India (IICB/IRB/2020/3P), in accordance with the Helsinki Declaration. The ARDS patients were recruited as part of a randomized control trial (CTRI/2020/05/025209) which has been completed and published as a preprint (15).

### Flow Cytometry

Plasma was isolated from the EDTA blood samples by centrifugation and cryostored. The whole-blood cell pellets were treated with 1 ml red blood cell (RBC) lysis buffer, and the RBC-depleted leukocytes were fixed with 1% paraformaldehyde before staining with the indicated fluorophore-tagged antibodies (BD Biosciences). The stained cells were acquired in a FACS ARIA III flow cytometer, and data were analyzed on FlowJo™ software.

### RNA Isolation From Nasopharyngeal Swab Samples and RT-PCR

RNA from nasopharyngeal swab samples in TRIzol was extracted using the chloroform-isopropanol method. RT-PCR for SARS-CoV-2 detection was performed using the STANDARD M nCoV Real-Time Detection kit (Cat No. 11NCO10, SD Biosensor), as per the manufacturer’s protocol. The kit suggested using the cutoff of Ct value 36 for the SARS-CoV-2 genes (RdRp and E gene) and the performance of the human positive control gene to declare a sample as SARS-CoV-2 positive. CY5-labeled Internal Control is used as a positive control. CT values are presented as average of the same for the two viral genes.

### SARS-CoV-2 Surrogate Virus Neutralization Assay

Neutralizing antibodies against SARS-CoV-2 in plasma samples from COVID-19 patients were detected using GenScript SARS-CoV-2 Surrogate Virus Neutralization kit (Cat no. L00847). Assay was performed according to the manufacturer’s protocol.

### Single-Cell RNA Sequencing Data Analysis

scRNA sequencing data were obtained from the publicly available GEO Datasets (accession numbers—GSE163668, GSE145926, and GSE168710 (5, 16, 17). For GSE163668, the study involved scRNA sequencing of a whole-blood sample of three patients with severe COVID-19 (GSM4995425) and two patients with mild/moderate COVID-19 (GSM4995426). For GSE145926, the study involved sequencing of all cells derived from the bronchoalveolar lavage fluid of three mild and six severe COVID-19 patients. For GSE168710, the study protocol depicted that isolated monocytes from the peripheral blood of four healthy donors were differentiated into macrophages with M-CSF treatment. Following this, the macrophages were cultured with IFN-β, IL-4, TNF-α, and IFN-γ, in combination or alone, in the presence or absence of synovial fibroblasts as indicated in the TSNE plots, before being subjected to scRNA sequencing. We analyzed the sequencing data from all three GEO datasets using the Seurat R package version 4.0 (18). The “LogNormalize” method was used for data normalization followed by identification of the top 4,000 variable features using the “vst” method and “FindVariableFeatures” function. Next, the “ScaleData” function was used for scaling the data. Principal component analysis was performed on the scaled data for dimensionality reduction using the “RunPCA” function, followed by clustering using the “FindNeighbours” and “FindClusters” functions. A TSNE plot of the data was generated using the “RunTSNE” function, and the “FeaturePlot” function was used to depict the expression of the indicated features on the TSNE plot. Finally, the target subset of interest characterized as HLA-DRA^low^ITGAX^high^ cells was selected and the expression of the “PLAUR” gene in these cells was visualized on the TSNE plot. The codes are available in the **Supplemental File**.

### Multiplex Cytokine Analysis

Plasma was isolated from peripheral blood of patients collected in EDTA vials. Cytokine levels (pg/ml) were measured in cryostored plasma using the Bio-Plex Pro Human Cytokine Screening Panel 48-Plex Assay (Bio-Rad, Cat No. 12007283), using the manufacturer’s protocol. Data for cytokines with detectable levels in at least 70% of the ARDS patients were analyzed and have been previously analyzed in the context of the convalescent plasma RCT, as noted before.

### ELISA for Soluble uPAR in Plasma

Soluble uPAR levels were measured in cryostored plasma using an ELISA kit for measuring the human protein (Human uPAR ELISA kit, Invitrogen, Cat no. EHPLAUR), following the manufacturer’s protocol. Quantitation of the plasma concentrations was derived from the OD values at 450 nm, measured on an ELISA plate reader (Bio-Rad), using a standard supplied by the manufacturer.

### Sample Preparation for Proteomics Analysis

Ten microliters of plasma was diluted to 100 μl with phosphate buffer, and protein precipitation was done by addition of 400 μl of acetone and incubation at 25°C for 2 min, followed by centrifugation at 10,000 g for 5 min. The pellets were air dried and suspended in 100 mM Tris–HCl buffer (pH 8.5). Protein estimation was performed using Bradford assay (Sigma-Aldrich, USA). For proteomics analysis, 20 μg of protein was reduced by addition of 25 mM of dithiothreitol (Sigma-Aldrich, USA) and incubated at 60°C for 30 min. Cysteine alkylation was performed by addition of 55 mM iodoacetamide (Sigma-Aldrich, USA) and incubated in the dark for 30 min at room temperature. Samples were digested with trypsin (V511A, Promega) with an enzyme-to-substrate ratio of 1:10 for 16 h at 37°C. The reaction was terminated with 0.1% formic acid and dried under vacuum. Peptide cleanup was done using an Oasis HLB 1-cc Vac cartridge (Waters).

### Mass Spectrometric Proteomics Analysis

DIA-SWATH analysis for the samples was done on a quadrupole-TOF hybrid mass spectrometer (TripleTOF 6600, Sciex, USA) coupled to a nano-LC system (Eksigent NanoLC 425). Four micrograms of these peptides was loaded on a trap column (ChromXP C18CL 5 μm 120 Å, Eksigent) where desalting was performed using 0.1% formic acid in water with a flow rate of 10 μl/min for 10 min. Peptides were separated on a reverse-phase C18 analytical column (ChromXP C18, 3 μm 120 Å, Eksigent) in a 57-min gradient of buffer A (0.1% formic acid in water) and buffer B (0.1% formic in acetonitrile) at a flow rate of 5 μl/min. Buffer B was slowly increased from 3% for 0 min to 25% in 38 min, further increased to 32% in the next 5 min, and ramped to 80% buffer B in the next 2 min. At 0.5 min, buffer B was increased to 90% and the column was washed for 2.5 min, buffer B was brought to initial 3% in the next 1 min, and the column was reconditioned for the next 8 min. The method with 100 precursor isolation windows was defined based on precursor m/z frequencies using the SWATH Variable Window Calculator (Sciex), with a minimum window of 5 m/z.

### Proteomics Data Analysis

Data were acquired using Analyst TF 1.7.1 Software (Sciex). Optimized source parameters were used. Ion spray voltage was set to 5.5 kV, 25 psi for the curtain gas, 35 psi for the nebulizer gas, and 250°C as source temperature. Accumulation time was set to 250 ms for the MS scan (400–1,250 m/z) and 25 ms for the MS/MS scans (100–1,500 m/z). Rolling collision energies were applied for each window based on the m/z range of each SWATH and a charge 2+ ion, with a collision energy spread of 5. The total cycle time was 2.8 s. The in-house spectral-ion library file (.group) was previously generated for human blood plasma proteins by searching.wiff format files generated in DDA mode against the UniProtKB human FASTA database (SWISS-PROT and TrEMBL; 74,255 entries) using ProteinPilot™ Software 5.0.1 (Sciex). A 1% global FDR at the protein level excluding shared peptides was considered for import in SWATH 2.0 MicroApp of PeakView 2.2 software (Sciex). SWATH run files were added, and retention time alignment was performed using peptides from abundant proteins. The processing settings for peak extraction were as follows: maximum of 10 peptides per protein, 5 transitions per peptide, >99% peptide confidence threshold, and 1% peptide FDR. The XIC extraction window was set to 10 min with 75 ppm XIC width. All information was exported in the form of MarkerView (.mrkw) files. In MarkerView 1.2.1 (Sciex), data normalization was performed using total area sum normalization and exported to excel. Data were deposited to the PRIDE database (19).

### Receiver Operator Characteristic Curve

The “cutpointr” package in R was used to generate the receiver operating characteristic (ROC) curve and calculate the corresponding AUC value for determining the suitability and dependability of suPAR content in the plasma of ARDS COVID patients as an indicator of their survival. The optimum threshold suPAR value which can be used to classify survival outcome in these patients with maximum sensitivity plus specificity was also determined.

### RNA-Seq Library Preparation and Sequencing

The RNA-Seq library was prepared using Illumina TruSeq Stranded Total RNA Library Prep Kit with Ribo-Zero Gold as per TruSeq Stranded Total RNA Reference Guide. We started with 250 ng of the total RNA. Briefly, the cytoplasmic and mitochondrial rRNA was depleted from the total RNA; the remaining RNA was purified, fragmented, and primed for cDNA synthesis. Subsequently, double-stranded cDNA (ds cDNA) was synthesized and the 3′ end of the ds cDNA was adenylated to provide an overhang for adapter ligation. The IDT for Illumina-TruSeq RNA UD Indexes was used for indexing the samples to allow multiplexing and then finally amplified and purified to enrich the adapter-ligated library. The final library was quantified using Qubit™ dsDNA High Sensitivity Assay Kit (Catalog number: Q32851), and the library size was determined using Agilent High Sensitivity DNA Kit (Catalog number: 5067–4626) on an Agilent Bioanalyzer 2100 platform. For sequencing, the individual library was diluted to 4 nM and libraries were pooled. The pooled library was denatured using 0.2 N NaOH, and neutralized with 200 mM Tris–HCl, pH 7.0. The libraries were sequenced on an Illumina NextSeq 550 platform, using high output Kit v2.5 (300 cycles), at a final library concentration of 1.6 pM (NextSeq 500 and NextSeq 550 Sequencing Systems: Denature and Dilute Libraries Guide; Document # 15048776 v16).

### RNA-Seq Data Processing

Filtered fastq files were processed using Salmon v1.4.0 which provides fast and bias-aware quantification of transcript expression (20). The mapping-based mode of Salmon was used for quantification (21). The reference transcriptome (Ensembl GRCh38, release 103) was used for indexing and quantification of the individual genes. The quantification was performed on the full transcriptome, and gene-level TPM values (transcripts per million) were computed based on the effective length of the transcripts. The TPM values are normalized for the gene length and sequencing depth and used for further analysis of differentially expressed genes.

### RNA-Seq Data Analysis

TPM values were analyzed using the online MeV software. The Limma tool (22) was used to find out the differentially expressed genes (DEGs) between the three different groups having three patients each, categorized according to the concentration of sUPAR in their plasma as described in the figure legend. The list of DEGs (transcripts) (p ≤ 0.05) provided by Limma was divided into two groups: (1) upregulated genes (having all differentially expressed transcripts upregulated) and (2) downregulated genes (having all differentially expressed transcripts downregulated) before being entered into the online NetworkAnalyst software (23) to obtain the list of enriched pathways (p ≤ 0.05) from the Reactome database, separately for upregulated and downregulated genes. Moreover, the genes specifically implicated in each of the pathways were also obtained.

### Correlation Matrix Generation and Visualization

A matrix of Spearman correlation coefficients indicating the degree and directionality of association between the concentrations of the indicated parameters in plasma of COVID-19 patients was generated using the “Hmisc” package in R. For visualization of the matrix, the “corrplot” package in R was used.

### Statistics

All statistical analyses, as depicted in the results as well in appropriate figures and their legends, were performed using GraphPad Prism 8 or in some cases using R. In all cases, Spearman correlation analysis and the Mann–Whitney test were performed unless otherwise stated. Primary outcomes of survival and disease remission (in terms of time till discharge from hospital) were compared between the two arms using Kaplan–Meier curve analysis—the Mantel–Haenszel hazard ratio was calculated, and statistical significance was tested by the Mantel–Cox log-rank test.

## Results

### Expansion of Circulating CD11c^+^HLA-DR^—^ Myeloid Cells Expressing suPAR in Severe COVID-19 Patients

**Figure 1:**
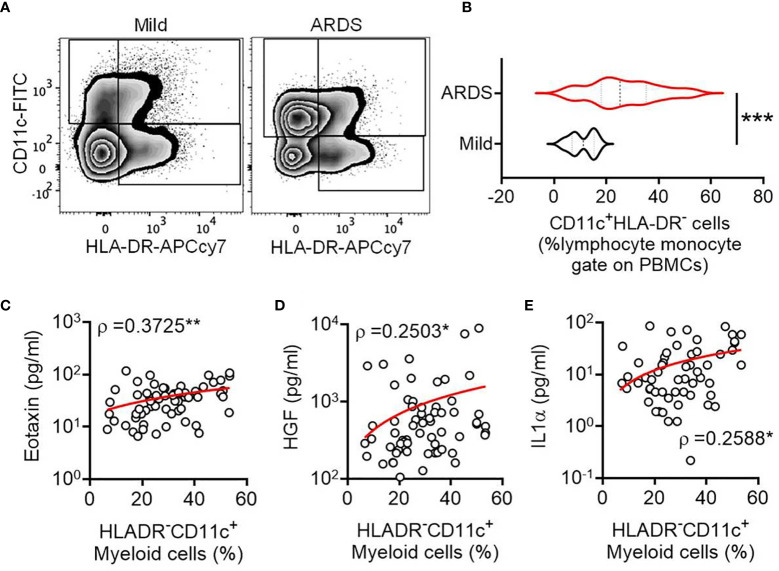
Expansion of circulating CD11c^+^HLA-DR^-^ myeloid cell subset in severe COVID-19. **(A)** Representative flow cytometry plots for gating of circulating CD11c^+^HLA-DR^-^ myeloid cells from COVID-19 patients with either mild disease or ARDS. **(B)** Violin plots showing the frequency of circulating CD11c^+^HLA-DR^-^ myeloid cells compared between COVID-19 patients with either mild disease or ARDS. The Mann Whitney test was performed, ***p < 0.0005. **(C–E)** Correlation between the frequency of circulating CD11c^+^HLA-DR^-^ myeloid cells and plasma level of the cytokines Eotaxin **(C)**, HGF **(D)**, and IL-1α **(E)** is plotted. Spearman ρ values are shown, **p < 0.005, *p < 0.05.

COVID-19 patients with mild symptoms (N = 16, age = 41.5 ± 18.95 years) or ARDS (N = 77, age = 61 ± 11.86 years) were recruited at the ID & BG Hospital, Kolkata, India (**Supplemental Table 1**). Peripheral blood sampling was done on the day of enrolment with due ethical approval from the institutional review board. The frequency of circulating CD11c^+^HLA-DR^–^ myeloid cells was assessed by flow cytometry. On comparing the relative abundance of circulating CD11c^+^HLA-DR^-^ proinflammatory macrophages between the COVID-19 patients with mild diseases and patients who progressed to ARDS, we found a prominent expansion of these cells in ARDS (**Figures 1A, B**), as reported by a number of previous studies as well (4–6). We found no correlation between abundance of these cells in circulation in ARDS patients and the plasma levels of most of the cytokines (among the 36 selected based on detectable levels in at least 70% of the ARDS patients in the cohort, data not shown), except for eotaxin (Pearson R = 0.3725, p = 0.0029), HGF (Pearson R = 0.2503, p = 0.0497), and IL-1α (Pearson R = 0.2588, p = 0.0423) (**Figures 1C–E**).

**Figure 2:**
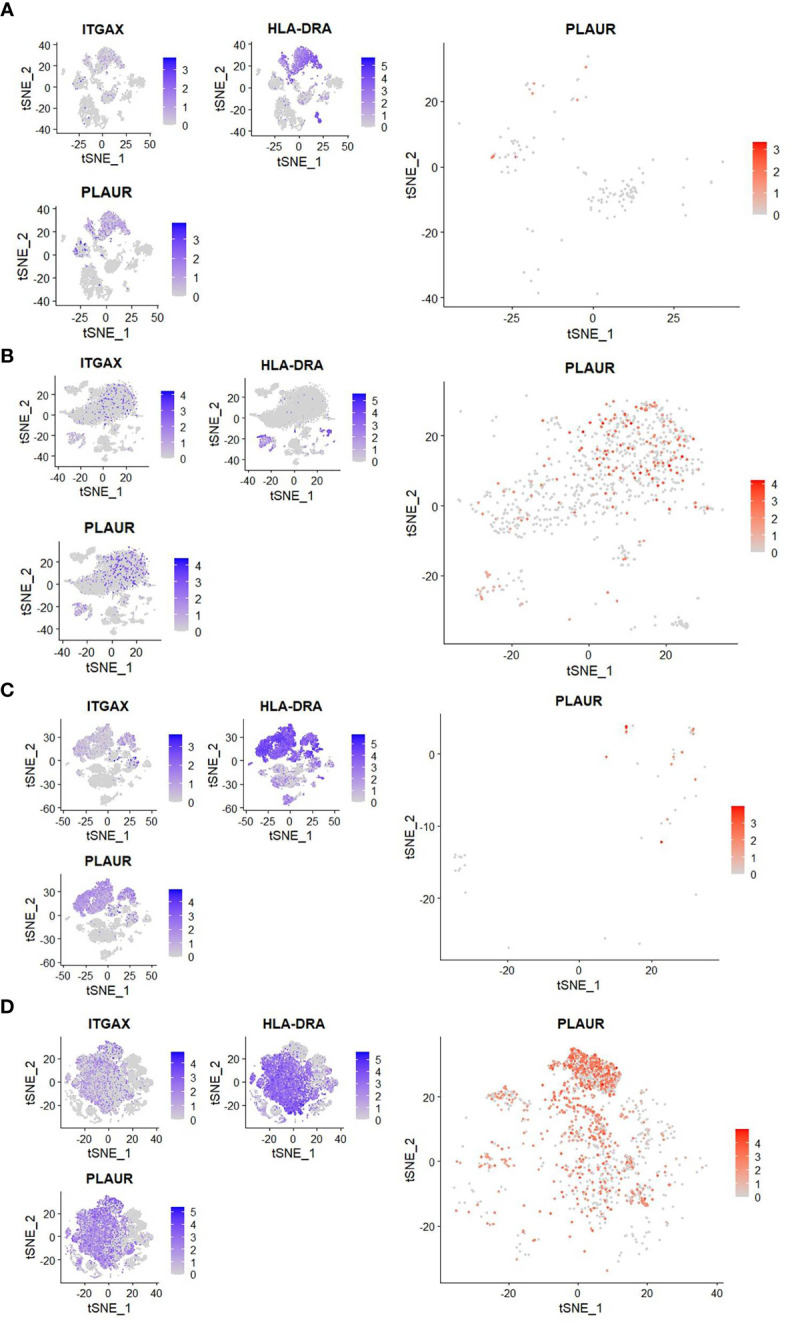
Reanalysis of single-cell sequencing data to confirm myeloid sourcing of suPAR. **(A, B)** Analysis of scRNAseq data (GSE163668) to compare the frequency of peripheral blood CD11c^high^HLA-DR^low^ cells and expression of PLAUR in them between patients with mild (left panel) or severe (right panel) COVID-19 disease. **(C, D)** Analysis of scRNAseq data (GSE GSE145926) to compare the frequency of CD11c^high^HLA-DR^low^ cells in the bronchoalveolar lavage fluid and the expression of PLAUR in them between patients with mild (left panel) or severe (right panel) COVID-19 disease. In both cases, three smaller plots on the left show distribution of expression of ITGAX (gene for CD11c), HLA-DRA (gene for HLA-DR), and PLAUR (gene for uPAR) among all cells. The bigger plot on the right shows expression of PLAUR among CD11c^high^HLA-DR^low^ cells.

Interestingly, a scRNAseq study reported an enriched expression of PLAUR, the gene for uPAR, in the similarly expanded myeloid cell compartment in severe COVID-19 (3). It intrigued us to explore if the circulating CD11c^+^HLA-DR^-^ myeloid cells in severe COVID-19 patients are enriched for uPAR expression. To this end, we analyzed two public datasets on scRNAseq, one done on whole blood (GSE163668, **Figures 2A, B**) and the other on cells in bronchoalveolar lavage fluid (GSE145926, **Figures 2C, D**) from COVID-19 patients with different disease states (5, 16). We found that the CD11c^high^HLA-DR^low^ myeloid cell subsets were highly abundant in patients with severe COVID-19 both in the circulation and in the airways on these analyses as well as noted a highly enriched expression of PLAUR in these CD11c^high^HLA-DR^low^ myeloid cells (**Figures 2B, D**).

### Higher Plasma Abundance of suPAR in Severe COVID-19 Is Linked to Systemic Cytokine Surge and TNFα-Activated Myeloid Cells

**Figure 3:**
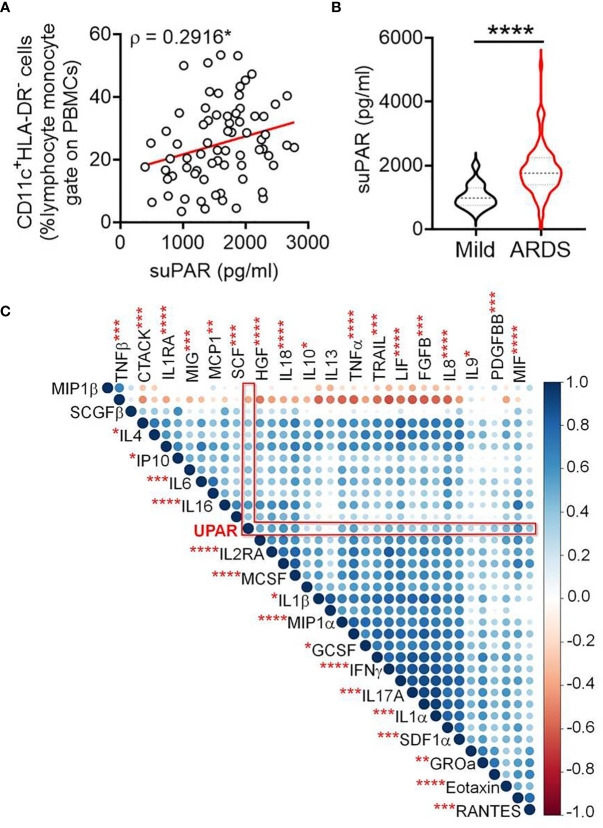
Increased plasma abundance of soluble uPAR and its cellular source in severe COVID-19. **(A)** Correlation between frequency of circulating CD11c^+^HLA-DR^–^ myeloid cells and plasma levels of suPAR in COVID-19 patients. **(B)** Plasma level of suPAR compared between COVID-19 patients with either mild disease or ARDS. **(C)** Corr plot showing mutual correlation between the plasma levels of 36 cytokines and soluble uPAR in COVID-19 patients with ARDS. The dots are color-coded and size-scaled for the Spearman ρ values for individual correlations; significance levels for correlation between plasma levels of suPAR and individual cytokines are noted as superscripts on cytokine names, ****p < 0.0001, ***p < 0.0005, **p < 0.005, and *p < 0.05.

Next, we measured the level of suPAR in plasma samples from the patients in our cohort and found a significant correlation between abundance of the circulating CD11c^+^HLA-DR^-^ myeloid cells and plasma suPAR levels (**Figure 3A**). Moreover, a significantly higher abundance of suPAR was noted in patients who have progressed to ARDS, compared to patients with milder symptoms (**Figure 3B**). The plasma level of suPAR had no relationship with either viral load of the ARDS patients at the time of plasma sampling (**Supplemental Figure 1A**) or the neutralizing antibody content of their plasma (**Supplemental Figure 1B**). Age or gender of the patients also did not show any effect (**Supplemental Figures 1C, D**).

**Figure 4:**
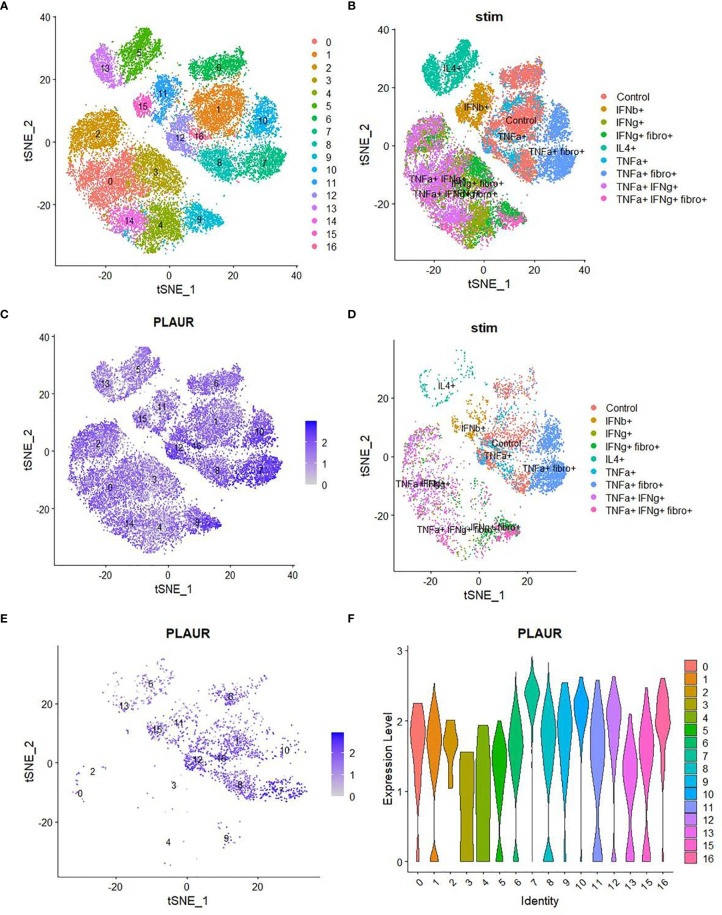
Analysis of single-cell sequencing data to confirm the role of TNFα on myeloid expression of suPAR. **(A)** TSNE plot of scRNA-seq data (obtained from GSE168710) of macrophages grouped into various clusters based on their gene expression profiles. **(B)** TSNE plot of the same scRNA-seq data showing the different treatments to which the macrophages were exposed before sequencing. **(C)** TSNE plot showing the distribution of expression of the “PLAUR” gene in all the cells of the same dataset. **(D)** TSNE plot depicting the treatment conditions to which all the PLAUR^high^ cells belong. **(E)** TSNE plot depicting the expression pattern of “PLAUR” specifically in CD11c^high^HLA-DR^low^ cells. **(F)** Violin plot highlighting the difference in expression of “PLAUR” between the different clusters of CD11c^high^HLA-DR^low^ cells.

The plasma level of suPAR was significantly correlated with plasma abundance of individual entities (in total 36 different cytokines), making up the systemic cytokine deluge in the ARDS patients (**Figure 3C**). This wide correlation of the plasma abundance of the cytokines with that of suPAR may mechanistically represent the effect of some of the proinflammatory cytokines on the expanded myeloid cells, inducing an expression of suPAR. On the other hand, it also presumably represents the amplified systemic inflammatory circuit leading to a correlated abundance. We noted a prominently significant correlation of plasma suPAR concentration with the plasma level of tumor necrosis alpha (TNFα), a major inflammatory cytokine capable of activating myeloid cells. In a recent study, monocyte-derived macrophages were stimulated with different cytokines and scRNAseq was performed to discover the heterogeneity of transcriptional responses (17). To shed some light on the mechanistic aspects of myeloid cell expression of suPAR, we analyzed this publicly available data (GSE168710) to look for the expression of PLAUR in these macrophages as they respond to different cytokine stimuli (**Figures 4A, B**). While the expression of PLAUR was noted in almost all the clusters of stimulated macrophages in this study (**Figure 4C**), stimulation with TNFα, in the absence of type I or type II interferons, drove the macrophages to achieve highest expression of PLAUR (**Figure 4D**). The CD11c^high^HLA-DR^low^ myeloid cells had a very high expression of PLAUR (**Figure 4E**), and again the highest expression was in response to TNFα (**Figure 4F**). Thus, TNFα, with its circulating levels, ramped up in the context of the systemic cytokine deluge, may play a key role in inducing suPAR expression in the abnormally expanded CD11c^high^HLA-DR^low^ myeloid cells in the severe ARDS patients, which warrants further mechanistic exploration.

### Linking Plasma suPAR Abundance and Inflammatory Plasma Proteome in Severe COVID-19

**Figure 5:**
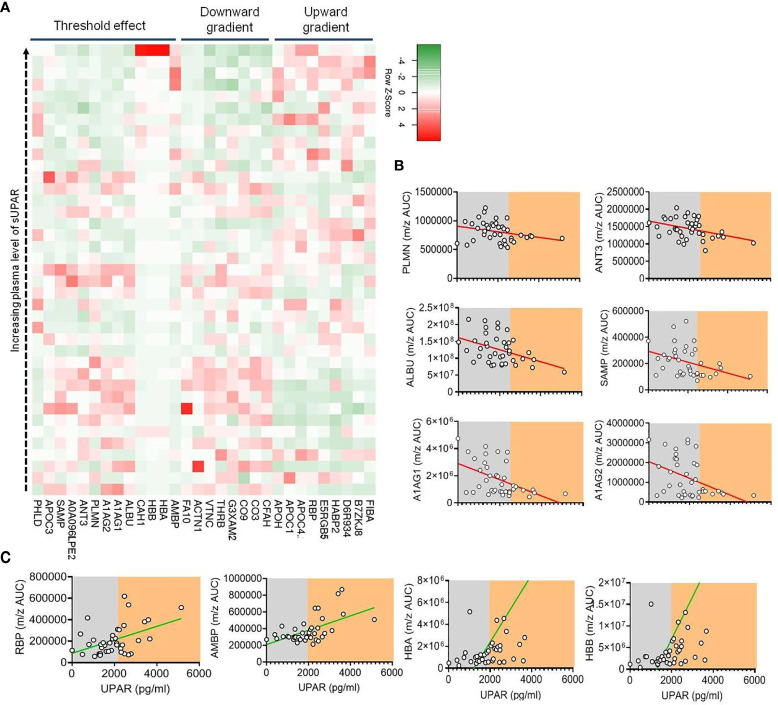
Proteomics analysis of plasma from patients with different plasma levels of suPAR. **(A)** Heatmap depicting the relation between the concentration of suPAR and the quantities of selected proteins (those which show significant correlations indicated, in plasma of Covid-19 patients). Each square represents one protein of one specific patient. The proteins have been categorized into three groups based on their pattern of expression—those which show a gradual increase with increase in suPAR, those which show a gradual decrease with increase in suPAR, and those which show sudden but significant change at a threshold plasma concentration of suPAR. **(B, C)** Representative line graphs for correlation between plasma suPAR concentration and area under curve of m/z peaks of proteins showing threshold downregulation **(B)**, viz., PLMN, ANT3, ALBU, SAMP, A1AG1, and A1AG2, and proteins showing threshold upregulation **(C)**, viz., RBP, AMBP, HBA, and HBB, to demonstrate the threshold effect, shown by arbitrary shaded demarcations.

As discussed, suPAR is functionally involved in the intricate regulation of both the coagulation cascade and complement activation. Thus, to glean further insights on the role of suPAR in severe COVID-19 pathogenesis, a plasma proteomics analysis was more insightful. We selected plasma samples across the range of different suPAR values (**Supplemental Figure 2A**). We could identify 179 proteins in our mass spectrometry-based study (**Supplemental Table 2**). The area under the curve for the m/z values of the respective peaks was used for a semiquantitative measure of abundance of those proteins in circulation. Plasma abundance of 24 proteins showed a statistically significant correlation with the plasma level of suPAR in the same plasma samples (**Supplemental Figure 2B**). A significant correlative clustering of these proteins was also apparent to variable extents. When we looked closely, the abundance of a number of these proteins showed a nice gradient, some increasing, the others decreasing, with increasing suPAR concentrations in plasma (**Figure 5A** and **Supplemental Table 3**).

The identities and functions of these proteins allowed us to appreciate a prominent signature of systemic hypercoagulability and complement activation (**Supplemental Table 3**). For example, increasing alpha fibrinogen (FIBA), hyaluronan-binding protein 2 (HABP2), and decreased abundance of plasminogen (PLMN), thrombin (THRB), factor X (FA10), anti-thrombin III (ANT3), and vitronectin (VTNC) point to systemic hypercoagulation associated with higher plasma suPAR levels (**Figure 5A** and **Supplemental Table 3**). On the other hand, complement C1q subunit B (D6R934) showing a positive correlation and complement factor 3 (CO3), vitronectin (VTNC), complement component 9 (CO9), complement factor H (CFAH), and complement factor I (G3XAM2) showing a negative correlation point to an increased complement activation state in patients with higher suPAR levels (**Figure 5A** and **Supplemental Table 3**).

Interestingly, we noted a prominent threshold state change for a number of proteins at a particular level of plasma suPAR abundance (**Figures 5B, C**). Notable among them were decrease in a few established anti-inflammatory acute-phase reactants and anti-coagulation factors like PLMN, ANT3, serum amyloid proteins (viz., SAMP), albumin (ALBU), and the alpha 1 acid glycoproteins (A1AG1 and A1AG2) and an increase in carbonic anhydrase 1 (CAH1), a metabolic enzyme known to have a proinflammatory effect on myeloid cells, retinol-binding protein (RBP) shown to play a role in inflammatory endotheliopathy, and hemoglobin alpha and beta chains (HBA and HBB) (**Figures 5B, C**).

### Identification of a Risk-Stratifying Cutoff for Plasma Level of suPAR Linked to Distinct Immune Transcriptome

**Figure 6:**
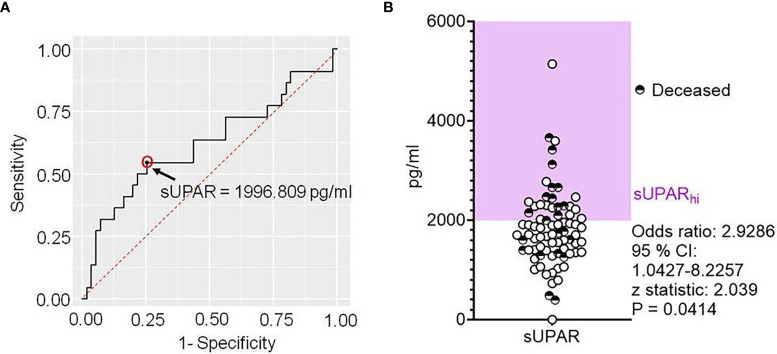
Deriving a cutoff for the plasma level of soluble uPAR linked to disease outcomes in severe COVID-19. **(A)** Receiver operator characteristic curve for plasma levels of suPAR as a predictor for fatal outcomes in severe COVID-19 patients. **(B)** Scatter plot showing the plasma level of suPAR for individual patients, also marking their final disease outcomes. The odds ratio was calculated for suPAR_hi_ patients to meet with fatal outcomes.

We were intrigued by the state-change pattern of these proteins at a certain threshold level of plasma suPAR level and wanted to explore if a cutoff value of the plasma suPAR concentration can be of interest for stratifying severe COVID-19 patients for clinical outcomes. We performed an ROC curve analysis for prediction of fatal outcomes of the disease. ROC analysis derived a cutoff value of 1,996.809 pg/ml (**Figure 6A**). Although sensitivity (54.55%) and specificity (74.55%) as well as area the under curve (0.619) on this analysis were not commensurate for suPAR being a prognostic biomarker, in a proportional odds analysis too, patients with suPAR levels higher than the cutoff value had a significantly higher odds ratio for meeting with fatal outcomes (**Figure 6B**).

**Figure 7:**
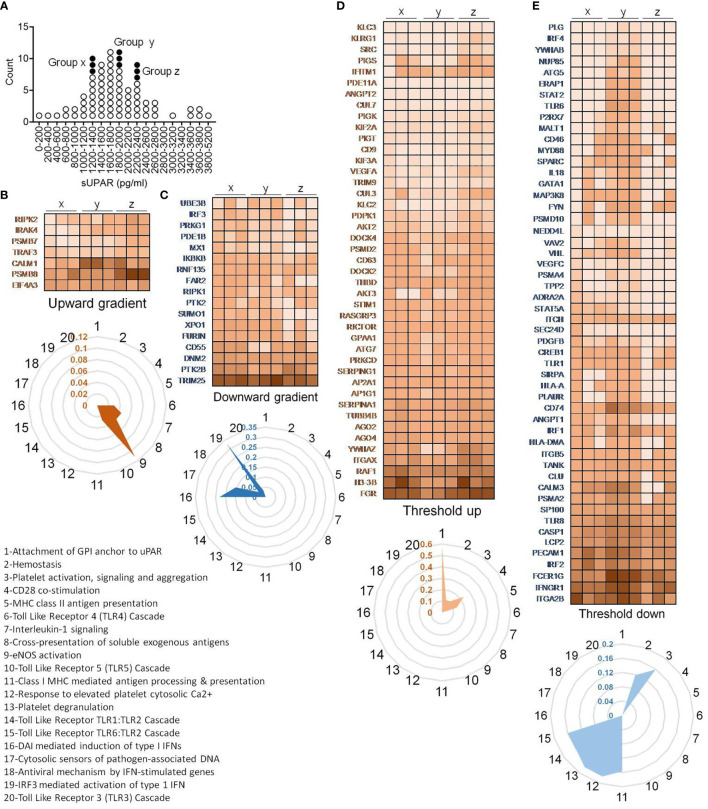
Peripheral blood transcriptome analysis of representative patients. **(A)** Plot showing distribution of patients and their selection for RNA sequencing based on their plasma concentration of suPAR. Each circle represents an individual patient, and the filled circles represent the samples selected for RNA sequencing. Three patients each were randomly selected from three distinct ranges of plasma suPAR values (three each in groups “x,” “y,” and “z,” total of nine patients) as indicated. Group “x” patients had low suPAR plasma levels, the group “y” patients had suPAR levels just below the cutoff, and the group “z” patients had plasma suPAR levels higher than the cutoff. **(B–E)** Heatmaps depicting changes in TPM values of selected differentially expressed genes. The genes belong to one of the four indicated groups, categorized on the basis of pattern of changes in expression in patients with increasing plasma suPAR levels. The genes belonging to the threshold up/downregulated categories include genes which show significant up/downregulation in the group z patients as compared to the group y patients, while either showing insignificant changes or significant changes in the opposite direction when comparing group y to group x. On the other hand, genes belonging to the gradient up/downregulated categories include those which show significant gradual/stepwise up/downregulation from group x to group z, through group y. The TPM value for each gene was calculated as the average of the TPM values of all the significant differentially expressed transcripts. Radar plots each depicting selected enriched pathways (for threshold/gradient up/downregulated genes) as determined from the Reactome database using the NetworkAnalyst software are shown below each heatmap **(B–E)**. The values represent the ratio of number of hits (genes) obtained in our dataset as compared to the total number of genes implicated in each pathway in the database. The pathways denoted as numbers (1–20) in the radar plots are listed.

To further explore if this cutoff value of plasma level of suPAR is mechanistically linked to gene expression patterns in the circulating immune cells, we performed a total RNA sequencing of peripheral blood cells. We selected nine representative patients for this analysis, three having low suPAR levels, three with plasma suPAR levels just below the cutoff value, and another three from patients with plasma suPAR values higher than the cutoff (**Figure 7A**).

In case of immune transcriptome, we also found that a subset of genes was differentially regulated showing an expression distribution in a gradient across the plasma suPAR concentrations (**Figures 7B, C**). Major pathways enriched for genes with upregulated expression with increasing suPAR concentration were signaling cascades for toll-like receptor (TLR) 4, TLR5 and IL1 receptor, pathway of cross-presentation of soluble exogenous antigens, and eNOS activation pathway (**Figure 7B**). The pathways enriched for genes with decreasing expression across the increasing suPAR gradient were mostly concerning type I interferon (IFN) responses to the virus, viz., pathways involving DNA-dependent activator of IFN-regulatory factors (DAI) pathway, TLR3 activation pathway, cytosolic DNA-sensing pathway, and pathways involved in antiviral mechanisms by IFN-stimulated genes and IRF3-mediated activation of type I IFNs (**Figure 7C**). Thus, the gene expression changes represented a deficiency in type I IFN-mediated antiviral mechanisms and ramped up systemic inflammation. The top panels in **Figures 7B, C** show the major genes that represent these pathways.

On the other hand, another subset of differentially expressed genes showed major changes across the cutoff value, which we called threshold upregulation or downregulation (**Figures 7D, E**). Major pathways enriched by this subset of upregulated genes were involved in uPAR signaling, coagulation cascade, and platelet functions (**Figure 7D**), confirming our insights gathered from the proteomics studies. It also included pathways involving T cell activation like antigen presentation by MHC class II and CD28 costimulatory pathways. Among the downregulated pathways were MHC class I antigen presentation, platelet degranulation, and the signaling cascade downstream of TLR1/2 and TLR6/2 (**Figure 7E**).

### Association of Plasma suPAR With Clinical Outcomes in Severe COVID-19

**Figure 8:**
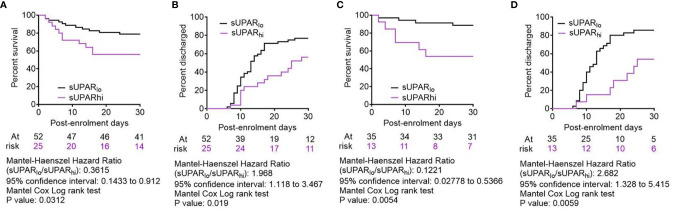
Comparison of clinical outcomes between severe COVID-19 patients with low and high plasma suPAR levels. **(A, C)** Survival of patients in the two arms from the day of enrolment till day 30 post-enrolment are compared in a Kaplan–Meier curve, for all age groups **(A)** as well as for patients aged <65 years **(C)**. Surviving patients were censored on day 30 post-enrolment. **(B, D)** Hospital stay duration of the patients from both groups (suPAR_lo_ and suPAR_hi_) since the day of enrolment is plotted in an ascending Kaplan–Meier curve, for all age groups **(B)** as well as for patients aged <65 years **(D)**. Deaths and non-remission at day 30 post-enrolment were censored. For all outcome comparisons, the Mantel–Cox log-rank test was performed.

To further validate the association of plasma suPAR level with the clinical outcomes in severe COVID-19 patients and its potential as a risk-stratifier, we compared the 30-day survival and time to disease remission (getting discharged from the hospital) between the ARDS patients with plasma suPAR levels lower than the derived cutoff value (designated suPAR_lo_) and the ones with higher than cutoff sUPAR levels (designated suPAR_hi_). The suPAR_lo_ patients were found to have significant survival benefit in Kaplan–Meier curve analysis as well as significantly faster remission, with median duration = 13 days for suPAR_lo_ patients compared to 25 days for the suPAR_hi_ group (**Figures 8A, B**).

Finally, we performed an extensive subclass analysis of the patients to get insight on better applicability of the suPAR cutoff for predicting survival in severe COVID-19 patients. First, we wanted to see if age of patients can influence the prediction efficacy, because aging has previously been shown to be a major deterrent for respiratory pathologies and worse response to therapy (24, 25). So we explored whether there was an enhanced survival benefit registered among younger patients in the suPAR_lo_ group. Previous studies have indicated significant differences in COVID-19 disease outcomes based on age of the patients (15, 26), perhaps due to inefficiency of an aging lung to mount regenerative response to tissue damage. Taking cue from these studies and also from a recent study identifying different age trajectories that represent resilience of human health in different age groups (27), we used an age cutoff of 65 years. It was found that the survival benefit of patients aged less than 65 years with low suPAR levels was way more significant, and they registered even faster remission (**Figures 8C, D**). We also performed subclass analyses between males and females among the ARDS patients (**Supplemental Figure 3A**), between patients who were diabetic or hypertensive and who were not (**Supplemental Figures 3B, C**), and patients who received different therapies, viz., corticosteroids, remdesivir, and convalescent plasma (**Supplemental Figures 3D–F**). While males, diabetics, normotensive patients, and patients receiving remdesivir as part of their therapies showed statistically significant survival benefits when they were suPAR_lo_, these subclass analyses were handicapped by lower sample sizes for the subclasses and thus warrant further exploration in bigger cohorts of severe COVID-19 patients.

## Discussion

Identification of high plasma levels of soluble uPAR in severe COVID-19 patients and association of lower plasma levels of suPAR with favorable clinical outcomes offer a strong risk stratifier, which will be of great translational value for effective triage and optimal timing for critical care in the patients. As discussed before, uPAR plays a regulatory role in both hemostatic pathways and complement pathway (10–13). In our study, insights gathered from proteomics and transcriptomics studies also pointed to the association of a state of hypercoagulation as well as complement activation with increasing suPAR levels. It was evident from the systemic depletion of coagulation factors like thrombin and factor X, the deficiency of regulatory proteins that inhibit coagulation, and a systemic dysbalance between factors that favor and inhibit complement activation. The decrease in plasma abundance of albumin may be indicative of an ongoing systemic or localized vascular leakage, which is known to be associated with the hypercoagulable states associated with systemic inflammations (28). The noted upward abundance across a threshold of the hemoglobin alpha and beta chains, which are normally not expected to be abundant in plasma, points to the possibility of a concomitantly active hemolytic mechanism. The state of increased complement activation can be linked to this possibility, although it warrants further mechanistic exploration. Notably, such occurrences are already reported in patients with COVID-19 (29–31). Moreover, suPAR has been shown to target the FPR1 receptor, expressed dominantly in neutrophils but also on other immune cells, playing a role in chemotactic migration as well as proinflammatory cytokine induction through signaling downstream of FPR1 (10). The interaction between uPAR and complement receptor 3 is known to regulate phagocytosis by neutrophils (13).

A previous study reporting higher suPAR plasma levels in critically ill patients found a healthy median plasma level of 2,100 pg/ml and also noted its dominant expression in myeloid cells (in this case neutrophils) (32). Similar steady-state plasma levels were also reported by studies noting higher suPAR levels in patients with COPD (33) and community-acquired pneumonia (34). Thus, the cutoff plasma level associated with favorable outcomes derived from the present Indian cohort of COVID-19 patients, which was 1,996.809 pg/ml, conforms to previous assessments in other cohorts. Of note here, a previous study with two small cohorts of severe COVID-19 patients from Greece and USA derived a cutoff of 6,000 pg/ml to be a strong predictor for progression to severe respiratory failure (14). Nevertheless, ethnic differences are expected to affect the non-pathogenic steady-state level of suPAR to a great extent and further studies are warranted in different ethnicities, which may also provide insights on variable susceptibility to COVID-19 severity. Moreover, differences in ELISA assays as well as assays based on proteomics, apart from the sampling source of the analyte, viz., plasma versus serum, may also lead to differences in terms of sensitivity and related parameters. Better risk stratification by plasma suPAR in patients aged less than 65 years from our cohort further highlights the relative deficiency in terms of resolution of tissue damage in aging lungs (24, 25).

Another important consideration for the potential of suPAR as a risk-stratifying biomarker is the long stability of suPAR in plasma samples, which makes it a useful biomarker for operational issues as well (35). Thus, the present study identified soluble uPAR as a useful biomarker for the prognostic stratification of COVID-19 patients who have progressed to ARDS in an Indian cohort. The ARDS pathophysiology in COVID-19 is increasingly being appreciated in terms of alveolar inflammation-associated pulmonary intravascular coagulation (36, 37). As uPAR-expressing myeloid cells are prevalent in both circulation and pulmonary tissue spaces, soluble uPAR may be a key link between the abnormally expanded circulating myeloid cell compartment in severe COVID-19 patients and the systemic hyper-inflammation and hypercoagulable state encountered in these patients, which warrants further mechanistic exploration.

## Data Availability Statement

The datasets presented in this study can be found in online repositories. The names of the repository/repositories and accession number(s) can be found in the article/**Supplementary Material**.

## Ethics Statement

The studies involving human participants were reviewed and approved by the Human Ethics committee of the ID & BG Hospital. The patients/participants provided their written informed consent to participate in this study.

## Author Contributions

JS, RD, PuB, MAH and BPS performed the ELISA, multiplex cytokine assay and flow cytometry; RD, PuB and SC did flow cytometric analysis; SRP, YR, KC, SB, DM, BS, BSS and PB recruited patients and participated in the clinical management of the patients; PS, MK and SSg performed the mass spectrometric proteomics; PM, SS, PD, PC and RP performed the next generation sequencing and analysis; DRC performed scRNAseq, RNAseq and ROC analysis; SP and PG helped with data analysis; DG conceptualized the study; DG and SSg designed the experiments; DG wrote the manuscript.

## Funding

The study was supported by grant no. MLP-129 from the Council of Scientific and Industrial Research (CSIR), India, to DG and partly supported by the Swarnajayanti Fellowship to DG. RNA-Seq was funded by the grants from CSIR (MLP-2005), Fondation Botnar (CLP-0031), IUSSTF (CLP-0033), and Intel (CLP-0034). DG is supported by Swarnajayanti Fellowship from the Department of Science & Technology, Govt. of India. JS, RD’R, and MH are supported by research fellowships from University Grants Commission, India. PB, PS, PD, and PC received research fellowships from CSIR. BPS received research fellowships from Indian Council of Medical Research, Government of India. PM and SS are supported by MLP-2005.

## Conflict of Interest

The authors declare that the research was conducted in the absence of any commercial or financial relationships that could be construed as a potential conflict of interest.

## Publisher’s Note

All claims expressed in this article are solely those of the authors and do not necessarily represent those of their affiliated organizations, or those of the publisher, the editors and the reviewers. Any product that may be evaluated in this article, or claim that may be made by its manufacturer, is not guaranteed or endorsed by the publisher.
