# Supplementary material for: Plasma Gradient of Soluble Urokinase-Type Plasminogen Activator Receptor Is Linked to Pathogenic Plasma Proteome and Immune Transcriptome and Stratifies Outcomes in Severe COVID-19
Source: Front Immunol. 2021 Oct 28;12:738093. doi: 10.3389/fimmu.2021.738093 (PMC8581406; doi:10.3389/fimmu.2021.738093)
Supplement: Supplementary file 1 [file DataSheet_1.pdf]

Plasma gradient of soluble urokinase-type plasminogen activator receptor is linked to pathogenic plasma proteome and immune transcriptome and stratifies outcomes in severe COVID-19

Jafar Sarif et al.

Supplemental information

- Supplemental figures with legends 1-3
- Supplemental table 1-4
- Supplemental computing codes
- Supplemental references 39-71

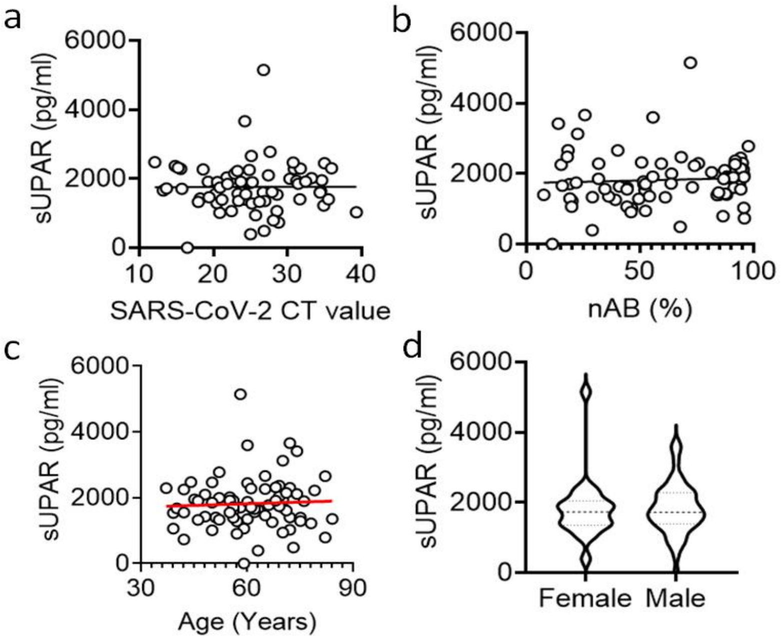

Supplemental figure 1

Supplemental figure 1. Correlation of plasma suPAR levels with specific parameters of the patients. (a) No correlation between average CT values for two SARS-CoV-2 genes and the plasma concentration of suPAR. (b) No correlation between percent neutralization function of patient plasma (derived from a surrogate assay measuring neutralization of receptor binding protein and ACE2 binding) and the plasma concentration of suPAR. (c) No correlation between age of patients with ARDS and the plasma level of suPAR. (d) Comparison between male and female patients with ARDS for the plasma level of suPAR. Mann Whitney test was performed.

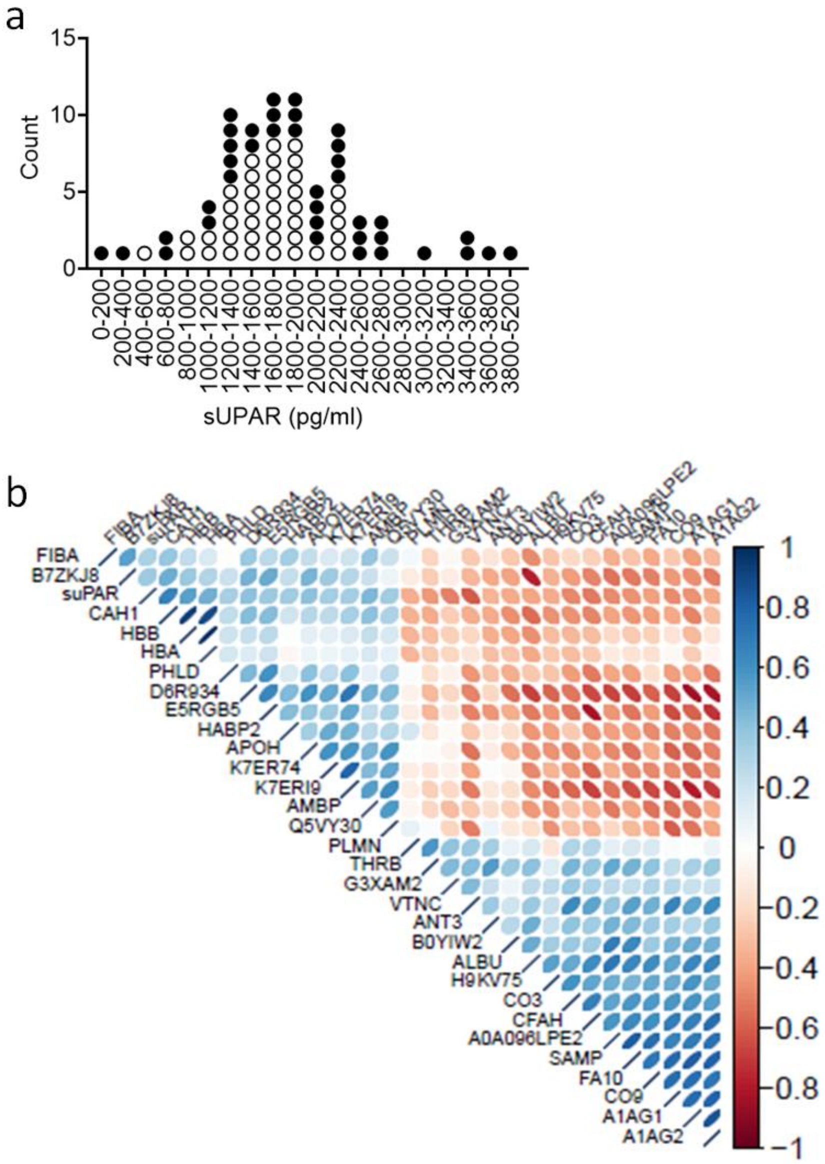

Supplemental figure 2

Supplemental figure 2. Plasma proteomics of representative patients and relationship with plasma level of suPAR. (a) Plot showing distribution of patients and their selection for proteomic studies based on their plasma concentration of suPAR. Each circle represents an individual patient and the filled circles represent the samples selected for mass-spectrometry based proteomic studies. Patients were randomly selected from distinct ranges of plasma suPAR values (total of 40 patients) as indicated, with the aim of being able to represent most of the ranges. (b) Corrplot depicting two-tailed Spearman correlation coefficients between the quantities of indicated proteins as well as suPAR in plasma of Covid-19 patients, generated using the 'Hmisc' package in R.

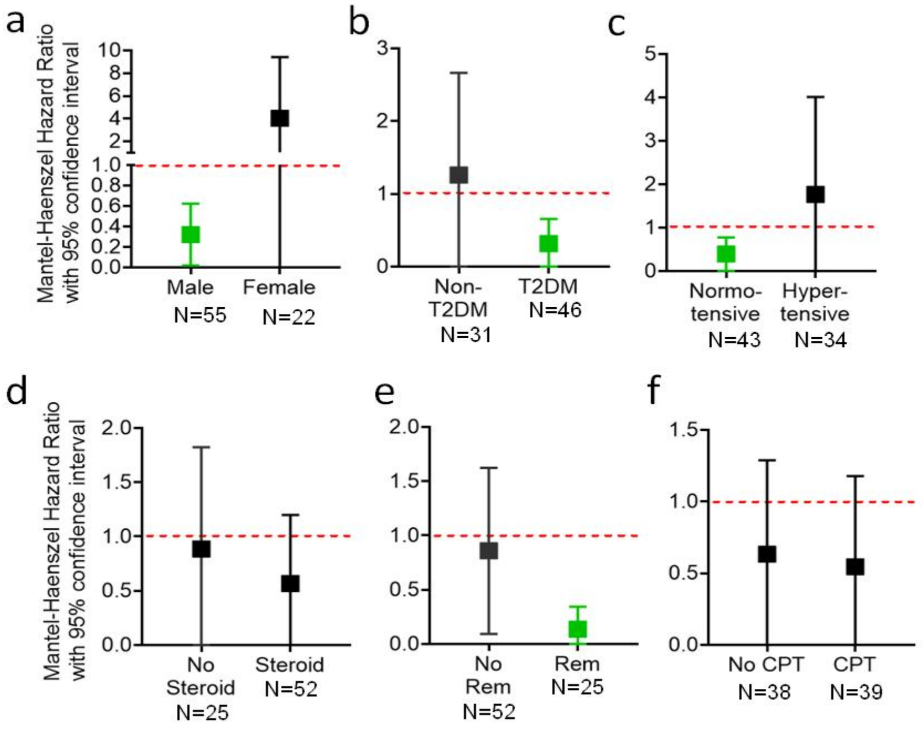

Supplemental figure 3

Supplemental figure 3. Subclass analysis for risk stratification of severe COVID-19 patients based on risk factors and received pharmacotherapies. (a) – (f) Forest plots of hazard ratio for survival benefits registered in $suPAR_{lo}$ patients, compared between males and females (a), patients with or without type 2 diabetes mellitus (b), patients with or without hypertension (c), treatment groups of corticosteroids (d), remdesivir (e) and convalescent plasma (f), across all age-groups. Deaths and non-remission at day 30 post-enrolment were censored. For all outcomes comparisons Mantel Cox log rank test was performed.

Supplemental table 1: Demography, co-morbidities and pharmacotherapy among the COVID-19 patients with ARDS

	**All ARDS**	**Low suPAR**	**High suPAR**
**Demography**			
Male	N=55, 71.43 %,<br>Age=62±12.12 years	N=36, 46.75%,<br>Age=59.5± 11.98 years	N=19, 24.68%,<br>Age=69±12.41 years
Female	N=22, 28.57%,<br>Age=59.5±10.03 years	N=16, 20.78%,<br>Age=59.5±10.22 years	N=6, 7.79%,<br>Age=59.5±10.45 years
**Major co-morbidities**			
Type 2 diabetes	N=46, 59.74%	N=33, 42.86%%	N13=, 16.88%
Hypertension	N=34, 44.16%	N=27, 35.06%	N=7, 9.09%
**Pharmacotherapy**			
Standard-of-care	N=77, 100%	N=52, 100%	N=25, 100%
Corticosteroids	N=52, 67.53%	N=39, 50.65%	N=13, 16.88%
Remdesivir	N=25, 32.47%	N=20, 25.97%	N=5, 6.49%
Convalescent plasma	N=39, 50.65%	N=26, 33.77%	N=13, 16.88%
**Renal function**			
Serum Creatinine	Available for N=57	N=42,<br>median 1.055mg/dl	N=15,<br>Median 1.14mg/dl

Supplemental table 2: List of proteins detected in the proteomics study on plasma from severe COVID-19 patients

Index	Peak Name	Group
1	sp|P02768|ALBU_HUMAN	Serum albumin OS=Homo sapiens OX=9606 GN=ALB PE=1 SV=2
2	sp|P04114|APOB_HUMAN	Apolipoprotein B-100 OS=Homo sapiens OX=9606 GN=APOB PE=1 SV=2
3	sp|P01024|CO3_HUMAN	Complement C3 OS=Homo sapiens OX=9606 GN=C3 PE=1 SV=2
4	sp|P01023|A2MG_HUMAN	Alpha-2-macroglobulin OS=Homo sapiens OX=9606 GN=A2M PE=1 SV=3
5	sp|P02787|TRFE_HUMAN	Serotransferrin OS=Homo sapiens OX=9606 GN=TF PE=1 SV=3
6	sp|P0C0L4|CO4A_HUMAN	Complement C4-A OS=Homo sapiens OX=9606 GN=C4A PE=1 SV=2
7	sp|P00450|CERU_HUMAN	Ceruloplasmin OS=Homo sapiens OX=9606 GN=CP PE=1 SV=1
8	sp|P01009|A1AT_HUMAN	Alpha-1-antitrypsin OS=Homo sapiens OX=9606 GN=SERPINA1 PE=1 SV=3
9	tr|A0A0A0MS08|A0A0A0MS08_HUMAN	Immunoglobulin heavy constant gamma 1 (Fragment) OS=Homo sapiens OX=9606 GN=IGHG1 PE=1 SV=1
10	sp|P02751|FINC_HUMAN	Fibronectin OS=Homo sapiens OX=9606 GN=FN1 PE=1 SV=4
11	sp|P02671|FIBA_HUMAN	Fibrinogen alpha chain OS=Homo sapiens OX=9606 GN=FGA PE=1 SV=2
12	sp|P08603|CFAH_HUMAN	Complement factor H OS=Homo sapiens OX=9606 GN=CFH PE=1 SV=4
13	sp|P02675|FIBB_HUMAN	Fibrinogen beta chain OS=Homo sapiens OX=9606 GN=FGB PE=1 SV=2
14	sp|P00738|HPT_HUMAN	Haptoglobin OS=Homo sapiens OX=9606 GN=HP PE=1 SV=1
15	tr|B4E1Z4|B4E1Z4_HUMAN	cDNA FLJ55673, highly similar to Complement factor B OS=Homo sapiens OX=9606 PE=1 SV=1
16	sp|P02679|FIBG_HUMAN	Fibrinogen gamma chain OS=Homo sapiens OX=9606 GN=FGG PE=1 SV=3
17	sp|P00747|PLMN_HUMAN	Plasminogen OS=Homo sapiens OX=9606 GN=PLG PE=1 SV=2
18	tr|B7ZKJ8|B7ZKJ8_HUMAN	ITIH4 protein OS=Homo sapiens OX=9606 GN=ITIH4 PE=1 SV=1
19	sp|P02774|VTDB_HUMAN	Vitamin D-binding protein OS=Homo sapiens OX=9606 GN=GC PE=1 SV=2
20	sp|P02647|APOA1_HUMAN	Apolipoprotein A-I OS=Homo sapiens OX=9606 GN=APOA1 PE=1 SV=1
21	sp|P01031|CO5_HUMAN	Complement C5 OS=Homo sapiens OX=9606 GN=C5 PE=1 SV=4
22	sp|P00734|THRB_HUMAN	Prothrombin OS=Homo sapiens OX=9606 GN=F2 PE=1 SV=2
23	tr|Q5T985|Q5T985_HUMAN	Inter-alpha-trypsin inhibitor heavy chain H2 OS=Homo sapiens OX=9606 GN=ITIH2 PE=1 SV=1
24	sp|P01871|IGHM_HUMAN	Immunoglobulin heavy constant mu OS=Homo sapiens OX=9606 GN=IGHM PE=1 SV=4
25	sp|P02790|HEMO_HUMAN	Hemopexin OS=Homo sapiens OX=9606 GN=HPX PE=1 SV=2
26	sp|P06727|APOA4_HUMAN	Apolipoprotein A-IV OS=Homo sapiens OX=9606 GN=APOA4 PE=1 SV=3
27	sp|P01876|IGHA1_HUMAN	Immunoglobulin heavy constant alpha 1 OS=Homo sapiens OX=9606 GN=IGHA1 PE=1 SV=2
28	sp|P04003|C4BPA_HUMAN	C4b-binding protein alpha chain OS=Homo sapiens OX=9606 GN=C4BPA PE=1 SV=2
29	sp|P01008|ANT3_HUMAN	Antithrombin-III OS=Homo sapiens OX=9606 GN=SERPINC1 PE=1 SV=1

30	sp|P01011|AACT_HUMAN	Alpha-1-antichymotrypsin OS=Homo sapiens OX=9606 GN=SERPINA3 PE=1 SV=2
31	sp|P68871|HBB_HUMAN	Hemoglobin subunit beta OS=Homo sapiens OX=9606 GN=HBB PE=1 SV=2
32	sp|P19827|ITIH1_HUMAN	Inter-alpha-trypsin inhibitor heavy chain H1 OS=Homo sapiens OX=9606 GN=ITIH1 PE=1 SV=3
33	sp|P10643|CO7_HUMAN	Complement component C7 OS=Homo sapiens OX=9606 GN=C7 PE=1 SV=2
34	sp|P06396|GELS_HUMAN	Gelsolin OS=Homo sapiens OX=9606 GN=GSN PE=1 SV=1
35	sp|P02765|FETUA_HUMAN	Alpha-2-HS-glycoprotein OS=Homo sapiens OX=9606 GN=AHSG PE=1 SV=2
36	sp|P01042|KNG1_HUMAN	Kininogen-1 OS=Homo sapiens OX=9606 GN=KNG1 PE=1 SV=2
37	sp|P04217|A1BG_HUMAN	Alpha-1B-glycoprotein OS=Homo sapiens OX=9606 GN=A1BG PE=1 SV=4
38	sp|P02749|APOH_HUMAN	Beta-2-glycoprotein 1 OS=Homo sapiens OX=9606 GN=APOH PE=1 SV=3
39	sp|P02649|APOE_HUMAN	Apolipoprotein E OS=Homo sapiens OX=9606 GN=APOE PE=1 SV=1
40	sp|P05155|IC1_HUMAN	Plasma protease C1 inhibitor OS=Homo sapiens OX=9606 GN=SERPING1 PE=1 SV=2
41	sp|P43652|AFAM_HUMAN	Afamin OS=Homo sapiens OX=9606 GN=AFM PE=1 SV=1
42	sp|P13671|CO6_HUMAN	Complement component C6 OS=Homo sapiens OX=9606 GN=C6 PE=1 SV=3
43	sp|P10909|CLUS_HUMAN	Clusterin OS=Homo sapiens OX=9606 GN=CLU PE=1 SV=1
44	tr|A0A0B4J231|A0A0B4J231_HUMAN	Immunoglobulin lambda-like polypeptide 5 OS=Homo sapiens OX=9606 GN=IGLL5 PE=1 SV=1
45	sp|P02763|A1AG1_HUMAN	Alpha-1-acid glycoprotein 1 OS=Homo sapiens OX=9606 GN=ORM1 PE=1 SV=1
46	tr|V9GYM3|V9GYM3_HUMAN	Apolipoprotein A-II OS=Homo sapiens OX=9606 GN=APOA2 PE=1 SV=1
47	sp|O75882|ATRN_HUMAN	Attractin OS=Homo sapiens OX=9606 GN=ATRN PE=1 SV=2
48	sp|P25311|ZA2G_HUMAN	Zinc-alpha-2-glycoprotein OS=Homo sapiens OX=9606 GN=AZGP1 PE=1 SV=2
49	sp|P04196|HRG_HUMAN	Histidine-rich glycoprotein OS=Homo sapiens OX=9606 GN=HRG PE=1 SV=1
50	sp|P02760|AMBP_HUMAN	Protein AMBP OS=Homo sapiens OX=9606 GN=AMBP PE=1 SV=1
51	sp|Q96PD5|PGRP2_HUMAN	N-acetylmuramoyl-L-alanine amidase OS=Homo sapiens OX=9606 GN=PGLYRP2 PE=1 SV=1
52	sp|P01834|IGKC_HUMAN	Immunoglobulin kappa constant OS=Homo sapiens OX=9606 GN=IGKC PE=1 SV=2
53	sp|P08697|A2AP_HUMAN	Alpha-2-antiplasmin OS=Homo sapiens OX=9606 GN=SERPINF2 PE=1 SV=3
54	sp|P20742|PZP_HUMAN	Pregnancy zone protein OS=Homo sapiens OX=9606 GN=PZP PE=1 SV=4
55	tr|A0A286YEY4|A0A286YEY4_HUMAN	Immunoglobulin heavy constant gamma 2 (Fragment) OS=Homo sapiens OX=9606 GN=IGHG2 PE=1 SV=1
56	tr|B4DPQ0|B4DPQ0_HUMAN	Complement C1r subcomponent OS=Homo sapiens OX=9606 GN=C1R PE=1 SV=1
57	sp|P09871|C1S_HUMAN	Complement C1s subcomponent OS=Homo sapiens OX=9606 GN=C1S PE=1 SV=1
58	sp|P02748|CO9_HUMAN	Complement component C9 OS=Homo sapiens OX=9606 GN=C9 PE=1

		SV=2
59	sp|P01019|ANGT_HUMAN	Angiotensinogen OS=Homo sapiens OX=9606 GN=AGT PE=1 SV=1
60	sp|P02766|TTHY_HUMAN	Transthyretin OS=Homo sapiens OX=9606 GN=TTR PE=1 SV=1
61	tr|G3XAM2|G3XAM2_HUMAN	Complement factor I OS=Homo sapiens OX=9606 GN=CFI PE=1 SV=1
62	sp|P02750|A2GL_HUMAN	Leucine-rich alpha-2-glycoprotein OS=Homo sapiens OX=9606 GN=LRG1 PE=1 SV=2
63	sp|P60709|ACTB_HUMAN	Actin, cytoplasmic 1 OS=Homo sapiens OX=9606 GN=ACTB PE=1 SV=1
64	sp|P01880|IGHD_HUMAN	Immunoglobulin heavy constant delta OS=Homo sapiens OX=9606 GN=IGHD PE=1 SV=3
65	sp|O43866|CD5L_HUMAN	CD5 antigen-like OS=Homo sapiens OX=9606 GN=CD5L PE=1 SV=1
66	tr|A0A087WW43|A0A087WW43_HUMAN	Inter-alpha-trypsin inhibitor heavy chain H3 OS=Homo sapiens OX=9606 GN=ITIH3 PE=1 SV=1
67	sp|P27169|PON1_HUMAN	Serum paraoxonase/arylesterase 1 OS=Homo sapiens OX=9606 GN=PON1 PE=1 SV=3
68	sp|P04004|VTNC_HUMAN	Vitronectin OS=Homo sapiens OX=9606 GN=VTN PE=1 SV=1
69	sp|Q08380|LG3BP_HUMAN	Galectin-3-binding protein OS=Homo sapiens OX=9606 GN=LGALS3BP PE=1 SV=1
70	sp|P36955|PEDF_HUMAN	Pigment epithelium-derived factor OS=Homo sapiens OX=9606 GN=SERPINF1 PE=1 SV=4
71	sp|P08185|CBG_HUMAN	Corticosteroid-binding globulin OS=Homo sapiens OX=9606 GN=SERPINA6 PE=1 SV=1
72	tr|H0YAC1|H0YAC1_HUMAN	Plasma kallikrein (Fragment) OS=Homo sapiens OX=9606 GN=KLKB1 PE=1 SV=1
73	sp|P05543|THBG_HUMAN	Thyroxine-binding globulin OS=Homo sapiens OX=9606 GN=SERPINA7 PE=1 SV=2
74	tr|A0A3B3ISJ1|A0A3B3ISJ1_HUMAN	Vitamin K-dependent protein S OS=Homo sapiens OX=9606 GN=PROS1 PE=1 SV=1
75	sp|P51884|LUM_HUMAN	Lumican OS=Homo sapiens OX=9606 GN=LUM PE=1 SV=2
76	tr|Q5VY30|Q5VY30_HUMAN	Retinol-binding protein OS=Homo sapiens OX=9606 GN=RBP4 PE=1 SV=2
77	sp|P22792|CPN2_HUMAN	Carboxypeptidase N subunit 2 OS=Homo sapiens OX=9606 GN=CPN2 PE=1 SV=3
78	tr|C9JF17|C9JF17_HUMAN	Apolipoprotein D (Fragment) OS=Homo sapiens OX=9606 GN=APOD PE=1 SV=1
79	sp|P35858|ALS_HUMAN	Insulin-like growth factor-binding protein complex acid labile subunit OS=Homo sapiens OX=9606 GN=IGFALS PE=1 SV=1
80	sp|P05546|HEP2_HUMAN	Heparin cofactor 2 OS=Homo sapiens OX=9606 GN=SERPIND1 PE=1 SV=3
81	tr|A0A286YES1|A0A286YES1_HUMAN	Immunoglobulin heavy constant gamma 3 (Fragment) OS=Homo sapiens OX=9606 GN=IGHG3 PE=1 SV=1
82	sp|P80108|PHLD_HUMAN	Phosphatidylinositol-glycan-specific phospholipase D OS=Homo sapiens OX=9606 GN=GPLD1 PE=1 SV=3
83	tr|J3KRP0|J3KRP0_HUMAN	Beta-Ala-His dipeptidase OS=Homo sapiens OX=9606 GN=CNDP1 PE=1 SV=2
84	sp|P00739|HPTR_HUMAN	Haptoglobin-related protein OS=Homo sapiens OX=9606 GN=HPR PE=2 SV=2
85	sp|P19652|A1AG2_HUMAN	Alpha-1-acid glycoprotein 2 OS=Homo sapiens OX=9606 GN=ORM2 PE=1 SV=2
86	tr|B0YIW2|B0YIW2_HUMAN	Apolipoprotein C-III OS=Homo sapiens OX=9606 GN=APOC3 PE=1 SV=1
87	tr|F5H7G1|F5H7G1_HUMAN	Complement component C8 beta chain OS=Homo sapiens OX=9606 GN=C8B PE=1 SV=1

88	sp|P69905|HBA_HUMAN	Hemoglobin subunit alpha OS=Homo sapiens OX=9606 GN=HBA1 PE=1 SV=2
89	sp|P07360|CO8G_HUMAN	Complement component C8 gamma chain OS=Homo sapiens OX=9606 GN=C8G PE=1 SV=3
90	sp|P05160|F13B_HUMAN	Coagulation factor XIII B chain OS=Homo sapiens OX=9606 GN=F13B PE=1 SV=3
91	sp|P07357|CO8A_HUMAN	Complement component C8 alpha chain OS=Homo sapiens OX=9606 GN=C8A PE=1 SV=2
92	sp|P01591|IGJ_HUMAN	Immunoglobulin J chain OS=Homo sapiens OX=9606 GN=JCHAIN PE=1 SV=4
93	tr|A0A286YFJ8|A0A286YFJ8_HUMAN	Immunoglobulin heavy constant gamma 4 (Fragment) OS=Homo sapiens OX=9606 GN=IGHG4 PE=1 SV=1
94	tr|E9PHK0|E9PHK0_HUMAN	Tetranectin OS=Homo sapiens OX=9606 GN=CLEC3B PE=1 SV=1
95	sp|P80748|LV321_HUMAN	Immunoglobulin lambda variable 3-21 OS=Homo sapiens OX=9606 GN=IGLV3-21 PE=1 SV=2
96	tr|A0A096LPE2|A0A096LPE2_HUMAN	SAA2-SAA4 readthrough OS=Homo sapiens OX=9606 GN=SAA2-SAA4 PE=4 SV=1
97	sp|P04264|K2C1_HUMAN	Keratin, type II cytoskeletal 1 OS=Homo sapiens OX=9606 GN=KRT1 PE=1 SV=6
98	tr|K7ER74|K7ER74_HUMAN	APOC4-APOC2 readthrough (NMD candidate) OS=Homo sapiens OX=9606 GN=APOC4-APOC2 PE=1 SV=1
99	sp|A0A0B4J1Y9|HV372_HUMAN	Immunoglobulin heavy variable 3-72 OS=Homo sapiens OX=9606 GN=IGHV3-72 PE=3 SV=1
100	sp|P43251|BTD_HUMAN	Biotinidase OS=Homo sapiens OX=9606 GN=BTD PE=1 SV=2
101	tr|A0A0J9YY99|A0A0J9YY99_HUMAN	Uncharacterized protein (Fragment) OS=Homo sapiens OX=9606 PE=1 SV=1
102	sp|P29622|KAIN_HUMAN	Kallistatin OS=Homo sapiens OX=9606 GN=SERPINA4 PE=1 SV=3
103	sp|O75636|FCN3_HUMAN	Ficolin-3 OS=Homo sapiens OX=9606 GN=FCN3 PE=1 SV=2
104	sp|O14791|APOL1_HUMAN	Apolipoprotein L1 OS=Homo sapiens OX=9606 GN=APOL1 PE=1 SV=5
105	sp|P04275|VWF_HUMAN	von Willebrand factor OS=Homo sapiens OX=9606 GN=VWF PE=1 SV=4
106	sp|P01619|KV320_HUMAN	Immunoglobulin kappa variable 3-20 OS=Homo sapiens OX=9606 GN=IGKV3-20 PE=1 SV=2
107	sp|P01599|KV117_HUMAN	Immunoglobulin kappa variable 1-17 OS=Homo sapiens OX=9606 GN=IGKV1-17 PE=1 SV=2
108	sp|P23083|HV102_HUMAN	Immunoglobulin heavy variable 1-2 OS=Homo sapiens OX=9606 GN=IGHV1-2 PE=1 SV=2
109	sp|P02747|C1QC_HUMAN	Complement C1q subcomponent subunit C OS=Homo sapiens OX=9606 GN=C1QC PE=1 SV=3
110	sp|P00915|CAH1_HUMAN	Carbonic anhydrase 1 OS=Homo sapiens OX=9606 GN=CA1 PE=1 SV=2
111	sp|P02042|HBD_HUMAN	Hemoglobin subunit delta OS=Homo sapiens OX=9606 GN=HBD PE=1 SV=2
112	sp|P00748|FA12_HUMAN	Coagulation factor XII OS=Homo sapiens OX=9606 GN=F12 PE=1 SV=3
113	tr|I3L145|I3L145_HUMAN	Sex hormone-binding globulin OS=Homo sapiens OX=9606 GN=SHBG PE=1 SV=1
114	sp|P01714|LV319_HUMAN	Immunoglobulin lambda variable 3-19 OS=Homo sapiens OX=9606 GN=IGLV3-19 PE=1 SV=2
115	sp|P01782|HV309_HUMAN	Immunoglobulin heavy variable 3-9 OS=Homo sapiens OX=9606 GN=IGHV3-9 PE=1 SV=2
116	sp|P0DOY2|IGLC2_HUMAN	Immunoglobulin lambda constant 2 OS=Homo sapiens OX=9606 GN=IGLC2 PE=1 SV=1

117	tr|H9KV75|H9KV75_HUMAN	Alpha-actinin-1 OS=Homo sapiens OX=9606 GN=ACTN1 PE=1 SV=1
118	sp|P02743|SAMP_HUMAN	Serum amyloid P-component OS=Homo sapiens OX=9606 GN=APCS PE=1 SV=2
119	tr|K7ERI9|K7ERI9_HUMAN	Apolipoprotein C-I (Fragment) OS=Homo sapiens OX=9606 GN=APOC1 PE=1 SV=1
120	sp|O95445|APOM_HUMAN	Apolipoprotein M OS=Homo sapiens OX=9606 GN=APOM PE=1 SV=2
121	sp|P00488|F13A_HUMAN	Coagulation factor XIII A chain OS=Homo sapiens OX=9606 GN=F13A1 PE=1 SV=4
122	sp|P01624|KV315_HUMAN	Immunoglobulin kappa variable 3-15 OS=Homo sapiens OX=9606 GN=IGKV3-15 PE=1 SV=2
123	sp|P04180|LCAT_HUMAN	Phosphatidylcholine-sterol acyltransferase OS=Homo sapiens OX=9606 GN=LCAT PE=1 SV=1
124	sp|A0A0C4DH38|HV551_HUMAN	Immunoglobulin heavy variable 5-51 OS=Homo sapiens OX=9606 GN=IGHV5-51 PE=3 SV=1
125	sp|P20851|C4BPB_HUMAN	C4b-binding protein beta chain OS=Homo sapiens OX=9606 GN=C4BPB PE=1 SV=1
126	sp|A0A0A0MS15|HV349_HUMAN	Immunoglobulin heavy variable 3-49 OS=Homo sapiens OX=9606 GN=IGHV3-49 PE=3 SV=1
127	sp|P00742|FA10_HUMAN	Coagulation factor X OS=Homo sapiens OX=9606 GN=F10 PE=1 SV=2
128	tr|D6R934|D6R934_HUMAN	Complement C1q subcomponent subunit B OS=Homo sapiens OX=9606 GN=C1QB PE=1 SV=1
129	sp|Q9NZP8|C1RL_HUMAN	Complement C1r subcomponent-like protein OS=Homo sapiens OX=9606 GN=C1RL PE=1 SV=2
130	tr|A0A087X0Q4|A0A087X0Q4_HUMAN	Immunoglobulin kappa variable 2-40 OS=Homo sapiens OX=9606 GN=IGKV2-40 PE=1 SV=1
131	sp|P0C0L5|CO4B_HUMAN	Complement C4-B OS=Homo sapiens OX=9606 GN=C4B PE=1 SV=2
132	sp|P01742|HV169_HUMAN	Immunoglobulin heavy variable 1-69 OS=Homo sapiens OX=9606 GN=IGHV1-69 PE=1 SV=2
133	tr|B1AHL2|B1AHL2_HUMAN	Fibulin-1 OS=Homo sapiens OX=9606 GN=FBLN1 PE=1 SV=1
134	sp|P01700|LV147_HUMAN	Immunoglobulin lambda variable 1-47 OS=Homo sapiens OX=9606 GN=IGLV1-47 PE=1 SV=2
135	sp|P01602|KV105_HUMAN	Immunoglobulin kappa variable 1-5 OS=Homo sapiens OX=9606 GN=IGKV1-5 PE=1 SV=2
136	tr|F8WF14|F8WF14_HUMAN	Carboxylic ester hydrolase OS=Homo sapiens OX=9606 GN=BCHE PE=1 SV=1
137	sp|P06312|KV401_HUMAN	Immunoglobulin kappa variable 4-1 OS=Homo sapiens OX=9606 GN=IGKV4-1 PE=1 SV=1
138	tr|B1AKG0|B1AKG0_HUMAN	Complement factor H-related protein 1 OS=Homo sapiens OX=9606 GN=CFHR1 PE=1 SV=1
139	tr|A0A0A0MRJ7|A0A0A0MRJ7_HUMAN	Coagulation factor V OS=Homo sapiens OX=9606 GN=F5 PE=1 SV=1
140	sp|P04433|KV311_HUMAN	Immunoglobulin kappa variable 3-11 OS=Homo sapiens OX=9606 GN=IGKV3-11 PE=1 SV=1
141	sp|P01772|HV333_HUMAN	Immunoglobulin heavy variable 3-33 OS=Homo sapiens OX=9606 GN=IGHV3-33 PE=1 SV=2
142	tr|E9PFZ2|E9PFZ2_HUMAN	Ceruloplasmin OS=Homo sapiens OX=9606 GN=CP PE=1 SV=1
143	sp|A0A0C4DH33|HV124_HUMAN	Immunoglobulin heavy variable 1-24 OS=Homo sapiens OX=9606 GN=IGHV1-24 PE=3 SV=1
144	sp|Q14520|HABP2_HUMAN	Hyaluronan-binding protein 2 OS=Homo sapiens OX=9606 GN=HABP2 PE=1 SV=1
145	sp|P04432|KVD39_HUMA	Immunoglobulin kappa variable 1D-39 OS=Homo sapiens OX=9606

	N	GN=IGKV1D-39 PE=3 SV=2
146	sp|P0DP07|HV431_HUMAN	Immunoglobulin heavy variable 4-31 OS=Homo sapiens OX=9606 GN=IGHV4-31 PE=3 SV=1
147	sp|P35908|K22E_HUMAN	Keratin, type II cytoskeletal 2 epidermal OS=Homo sapiens OX=9606 GN=KRT2 PE=1 SV=2
148	sp|P13645|K1C10_HUMAN	Keratin, type I cytoskeletal 10 OS=Homo sapiens OX=9606 GN=KRT10 PE=1 SV=6
149	sp|P08519|APOA_HUMAN	Apolipoprotein(a) OS=Homo sapiens OX=9606 GN=LPA PE=1 SV=1
150	sp|P01721|LV657_HUMAN	Immunoglobulin lambda variable 6-57 OS=Homo sapiens OX=9606 GN=IGLV6-57 PE=1 SV=2
151	sp|P06681|CO2_HUMAN	Complement C2 OS=Homo sapiens OX=9606 GN=C2 PE=1 SV=2
152	sp|P08571|CD14_HUMAN	Monocyte differentiation antigen CD14 OS=Homo sapiens OX=9606 GN=CD14 PE=1 SV=2
153	sp|O95497|VNN1_HUMAN	Pantetheinase OS=Homo sapiens OX=9606 GN=VNN1 PE=1 SV=2
154	tr|G3V0E5|G3V0E5_HUMAN	Transferrin receptor (P90, CD71), isoform CRA_c OS=Homo sapiens OX=9606 GN=TFRC PE=1 SV=1
155	tr|Q5VVP7|Q5VVP7_HUMAN	C-reactive protein OS=Homo sapiens OX=9606 GN=CRP PE=1 SV=1
156	sp|A0A0C4DH69|KV109_HUMAN	Immunoglobulin kappa variable 1-9 OS=Homo sapiens OX=9606 GN=IGKV1-9 PE=3 SV=1
157	tr|A0A087WXI2|A0A087WXI2_HUMAN	IgGFc-binding protein (Fragment) OS=Homo sapiens OX=9606 GN=FCGBP PE=1 SV=2
158	tr|A0A024R6I7|A0A024R6I7_HUMAN	Alpha-1-antitrypsin OS=Homo sapiens OX=9606 GN=SERPINA1 PE=1 SV=1
159	sp|A0A075B6K5|LV39_HUMAN	Immunoglobulin lambda variable 3-9 OS=Homo sapiens OX=9606 GN=IGLV3-9 PE=3 SV=1
160	sp|A0A0C4DH68|KV224_HUMAN	Immunoglobulin kappa variable 2-24 OS=Homo sapiens OX=9606 GN=IGKV2-24 PE=3 SV=1
161	sp|A0M8Q6|IGLC7_HUMAN	Immunoglobulin lambda constant 7 OS=Homo sapiens OX=9606 GN=IGLC7 PE=1 SV=3
162	sp|A0A0B4J1X5|HV374_HUMAN	Immunoglobulin heavy variable 3-74 OS=Homo sapiens OX=9606 GN=IGHV3-74 PE=3 SV=1
163	tr|A0A0C4DH35|A0A0C4DH35_HUMAN	Immunoglobulin heavy variable 3-35 (non-functional) (Fragment) OS=Homo sapiens OX=9606 GN=IGHV3-35 PE=1 SV=1
164	tr|A0A0G2JSC0|A0A0G2JSC0_HUMAN	Immunoglobulin lambda variable 5-45 (Fragment) OS=Homo sapiens OX=9606 GN=IGLV5-45 PE=1 SV=1
165	tr|A0A0G2JMB2|A0A0G2JMB2_HUMAN	Immunoglobulin heavy constant alpha 2 (Fragment) OS=Homo sapiens OX=9606 GN=IGHA2 PE=1 SV=1
166	sp|A0A075B6K4|LV310_HUMAN	Immunoglobulin lambda variable 3-10 OS=Homo sapiens OX=9606 GN=IGLV3-10 PE=3 SV=2
167	sp|P01766|HV313_HUMAN	Immunoglobulin heavy variable 3-13 OS=Homo sapiens OX=9606 GN=IGHV3-13 PE=1 SV=2
168	sp|P01743|HV146_HUMAN	Immunoglobulin heavy variable 1-46 OS=Homo sapiens OX=9606 GN=IGHV1-46 PE=1 SV=2
169	sp|A0A0C4DH31|HV118_HUMAN	Immunoglobulin heavy variable 1-18 OS=Homo sapiens OX=9606 GN=IGHV1-18 PE=3 SV=1
170	tr|A0A075B7D0|A0A075B7D0_HUMAN	Immunoglobulin heavy variable 1/OR15-1 (non-functional) (Fragment) OS=Homo sapiens OX=9606 GN=IGHV1OR15-1 PE=1 SV=1
171	tr|E9PQD6|E9PQD6_HUMAN	Serum amyloid A protein OS=Homo sapiens OX=9606 GN=SAA1 PE=1 SV=1
172	tr|A0A2Q2TTZ9|A0A2Q2TTZ9_HUMAN	Immunoglobulin kappa variable 1-33 OS=Homo sapiens OX=9606 GN=IGKV1D-33 PE=1 SV=1
173	tr|A0A075B7B8|A0A075B	Immunoglobulin heavy variable 3/OR16-12 (non-functional)

	7B8_HUMAN	(Fragment) OS=Homo sapiens OX=9606 GN=IGHV3OR16-12 PE=1 SV=1
174	sp|A0A0C4DH34|HV428_HUMAN	Immunoglobulin heavy variable 4-28 OS=Homo sapiens OX=9606 GN=IGHV4-28 PE=3 SV=1
175	tr|H0YCQ7|H0YCQ7_HUMAN	Glutamine and serine-rich protein 1 (Fragment) OS=Homo sapiens OX=9606 GN=QSER1 PE=1 SV=1
176	tr|E5RGB5|E5RGB5_HUMAN	Bridging integrator 3 OS=Homo sapiens OX=9606 GN=BIN3 PE=4 SV=1
177	tr|C9J6K0|C9J6K0_HUMAN	Secreted phosphoprotein 24 (Fragment) OS=Homo sapiens OX=9606 GN=SPP2 PE=1 SV=1
178	tr|A0A2R8YGX3|A0A2R8YGX3_HUMAN	Tropomyosin alpha-4 chain OS=Homo sapiens OX=9606 GN=TPM4 PE=1 SV=1
179	sp|Q9HAK2|COE2_HUMAN	Transcription factor COE2 OS=Homo sapiens OX=9606 GN=EBF2 PE=2 SV=4

Supplemental table 3: Proteins showing significantly correlated plasma abundance with plasma suPAR concentrations

Protein	Correlation with plasma suPAR concentration	Functional attributes and potential role in pathology	Projected role in disease outcome
FIBA	Monotonic, positive	FIBA or alpha fibrinogen, on cleaved by thrombin generates fibrin, the most abundant protein in a blood clot.	Unfavorable
D6R934 (C1QB)	Monotonic, positive	C1QB or Complement C1Q subunit B, is a critical component of the classical complement pathway, thus has both tissue-sparing as well as tissue damaging effects depending on contexts of infections, it has been shown to upregulated in myeloid cells in COVID-19 patients [39].	Unfavorable
PHLD	Threshold effect, positive	Phosphatidylinositol-glycan-specific phospholipase D hydrolyses GPI-anchors of several inflammatory proteins upon hydrolysis of proinflammatory cytokines like IL-1, TNF-α [40]	Unfavorable
HABP2	Monotonic, positive	HABP2 or Hyaluronan-binding protein 2, also known as FSAP or factor VII activating protease is a critical coagulation factor [41, 42]. Increased abundance of HABP2 is known in sepsis [43].	Unavorable
B7ZKJ8 (ITIH4)	Monotonic, positive	ITIH4 or Inter-alpha-trypsin inhibitor heavey chain 4, is upregulated in inflammatory contexts, mainly in response to IL-6 signaling [44]. It has also shown to upregulate in COPD [45]. Its anti-inflammatory role is evident from its protease inhibitory function and inhibition of MASP-1 and plasma kallikrein [46].	Favorable
APOH	Monotonic, positive	APOH or Apolipoprotein H, also known as beta-2 glycoprotein 1, plays a role in coagulation [47, 48] with predominantly anti-coagulatory function, has been shown to increase in abundance in inflammatory contexts like COPD and SLE.	Favorable
K7ERI9 and K7ER74 (APOC1 and APOC4-2)	Monotonic, positive	Apolipoprotein C1 as well as C4 and C2 associates with triglyceride-rich lipoproteins and HDL and thus is critical in lipid metabolism and play a role in antiinflamatory role of HDL [49]. In addition APOC1 is known to be reduced in sepsis associated inflammation and shown to seclude bacterial TLR4 ligands in such contexts [50, 51].	Favorable
E5RGB5 (BIN3)	Monotonic, positive	BIN3 or Bridging integrator 3, is a protein implicated in endocytic pathways [52].	Favorable?
CAH1	Threshold effect,	CAH1 or Carbonic anhydrase 1 is member of a	Unfavorable

	positive	ubiquitously expressed family of enzymes that catalyze reversible hydration of carbon dioxide to bicarbonate and protons. Interestingly a pro-inflammatory role of CAH is known in macrophages as well as in mast cells [53, 54].	
RBP	Threshold effect, positive	Retinol binding protein 4 is a member of lipocalin superfamily helps in inducing proinflammatory molecule expression and also helps in lymphocyte recruitment and adherence to the endothelium by stimulating human retinal capillary endothelial cells (HRCEC) and human umbilical vein endothelial cells (HUVEC) [55].	Unfavorable
AMBP	Threshold effect, positive	AMBP or Alpha-1-Microglobulin/Bikunin Precursor, an anti-inflammatory proteoglycan that stabilizes extracellular matrix by interacting with hyluronan, in combination with inter-alpha-trypsin inhibitor (ITIH1) [56, 57].	Favorable?
HBA	Threshold effect, positive	Hemoglobin alpha chain, plasma hemoglobin may be indicative of a haemolytic process.	Unfavorable Hemolytic anemia?
HBB	Threshold effect, positive	Hemoglobin beta chain, plasma hemoglobin may be indicative of a haemolytic process.	Unfavorable Hemolytic anemia?
CO3	Monotonic, negative	CO3 or Complement 3, is a key component for both classical and alternative pathways of complement activation. Proteolytic cleavage of C3 leads to the active forms which play roles in opsonization and anaphylaxis. Reduction in C3 levels has been found to be linked with worse outcomes in COVID-19 [58].	Unfavorable
CO9	Monotonic, negative	CO9 or complement component 9, is a major component of the membrane attack complex (MAC) formed as the final outcome of complement activation. Depletion of plasma CO9 may indicate its increased consumption due to recruitment of it in cell-surface MACs [59].	Unfavorable
CFAH	Monotonic, negative	CFAH or complement factor H, is a regulatory glycoprotein for complement activation which prevents host cell damage by binding to C3 [60].	Unfavorable
G3XAM2 (CFI)	Monotonic, negative	G3XAM2 (CFI) or Complement factor I, inhibits complement activation by cleaving C3b and C4b [61]. Genetic abnormalities of CFI have been associated with complement mediated hemolysis [62].	Unfavorable
THRB	Monotonic, negative	THRB or Thrombin, is a serine protease which drives fibrinogen to fibrin conversion thus being critical for coagulation. Depletion of circulating thrombin may indicate systemic	Unfavorable

		coagulopathy associated with COVID-19 [63].	
FA10	Monotonic, negative	FA10 or Factor X is the critical regulator of coagulation cascade which cleaves prothrombin to generate thrombin. Depletion of circulating FA10 may result from systemic coagulopathy associated with COVID-19.	Unfavorable
VTNC	Monotonic, negative	VTNC or Vitronectin is a circulating glycoprotein playing critical roles in both inhibition of coagulopathy, through inhibition of plasmaminogen activator inhibitor (PAI) as well as by protecting against complment-mediated cellular damage [64, 65].	Unfavorable
ACTN1	Monotonic, negative	ACTN1 or alpha actinin 1 is a cytoskeletal protein associated with cellular junctions with critical physiological effects in smooth muscle cells as well as epithelia and endothelial cells. Plasma level of ACTN1 may indicate smooth muscle cell injury including injury to myocardium resulting from systemic inflammation.	Favorable?
A1AG1 and A1AG2	Threshold effect, negative	Alpha 1 acid glycoprotein 1 and 2, are acute phase reactants produced by liver and peripheral tissues in response to systemic inflammation and has been shown to regulate inflammatory cells and mediators with an anti-inflammatory and anti-fibrogenic role [66, 67].	Unfavorable?
PLMN	Threshold effect, negative	PLMN or plasminogen is the precursor for the fibrinolytic protein plasmin. Deficiency of peptides constituent of plasminogen may point to deficiency of the fibrinolytic process leading to systemic coagulopathy, established to be associated with COVID-19 [68].	Unfavorable
ANT3	Threshold effect, negative	ANT3 or antithrombin III, is a glycoprotein with regulatory role on intrinsic pathway of coagulation mediated by inhibition of the proteases taking part in the coagulation cascade [69].	Unfavorable
ALBU	Threshold effect, negative	ALBU or Albumin, depletion from plasma is indicative of vascular leakage during systemic inflammation. It has been shown to be associated with hypercoagulable state associated with ANT3 reduction encountered in systemic inflammation [70].	Unfavorable
SAMP	Threshold effect, negative	SAMP or Serum amyloid P is an acute phase reactant of the pantraxin family known to have anti-inflammatory as well as anti-fibrogenic roles [71].	Unfavorable
A0A096LPE2	Threshold effect, negative	SAA2-SAA4 readthrough, serum amyloid A proteins, are acute phase reactants induced in response to systemic inflammation, which	Unfavorable

		help in immune cell recruitment.	
APOC3	Threshold effect, negative	APOC3 or apolipoprotein C3 is associated with VLDL particles and an increased level portends hypertriglyceridemia.	Favorable

Supplemetal table 4. Pathways enriched by differentially expressed genes between group 'x', 'y' and 'z' patients.

A. Enriched pathways for gradient upregulated genes

Pathway	Total	Expected	Hits	P.Value	FDR
Destabilization of mRNA by AUF1 (hnRNP D0)	54	0.259	3	0.0021	0.813
The citric acid (TCA) cycle and respiratory electron transport	145	0.695	4	0.00478	0.813
Deadenylation of mRNA	24	0.115	2	0.00575	0.813
Smooth Muscle Contraction	25	0.12	2	0.00623	0.813
Respiratory electron transport	82	0.393	3	0.00685	0.813
p75 NTR receptor-mediated signalling	85	0.407	3	0.00757	0.813
Regulation of mRNA Stability by Proteins that Bind AU-rich Elements	88	0.422	3	0.00833	0.813
Interactions of Tat with host cellular proteins	2	0.00959	1	0.00956	0.813
Synthesis of DNA	95	0.455	3	0.0103	0.813
Activated TLR4 signalling	100	0.479	3	0.0118	0.813
Respiratory electron transport, ATP synthesis by chemiosmotic coupling, and heat production by uncoupling proteins.	101	0.484	3	0.0121	0.813
DNA Replication	102	0.489	3	0.0125	0.813
Toll Like Receptor 4 (TLR4) Cascade	103	0.494	3	0.0128	0.813
Metabolism of mRNA	317	1.52	5	0.0164	0.813
HIV Infection	214	1.03	4	0.0183	0.813
Axonal growth stimulation	4	0.0192	1	0.019	0.813
Activation of CaMK IV	4	0.0192	1	0.019	0.813
Interleukin-1 signaling	45	0.216	2	0.0194	0.813
S Phase	122	0.585	3	0.0201	0.813
Toll-Like Receptors Cascades	123	0.589	3	0.0205	0.813
Rho GTPase cycle	123	0.589	3	0.0205	0.813
Signaling by Rho GTPases	123	0.589	3	0.0205	0.813
Metabolism of RNA	339	1.62	5	0.0213	0.813
Regulation of activated PAK-2p34 by proteasome mediated degradation	48	0.23	2	0.0219	0.813
Cross-presentation of soluble exogenous antigens	48	0.23	2	0.0219	0.813

(endosomes)					
Ubiquitin-dependent degradation of Cyclin D1	49	0.235	2	0.0227	0.813
CDK-mediated phosphorylation and removal of Cdc6	49	0.235	2	0.0227	0.813
Ubiquitin-dependent degradation of Cyclin D	49	0.235	2	0.0227	0.813
Regulation of ornithine decarboxylase (ODC)	49	0.235	2	0.0227	0.813
Vpu mediated degradation of CD4	50	0.24	2	0.0236	0.813
CaMK IV-mediated phosphorylation of CREB	5	0.024	1	0.0237	0.813
Cell Cycle Checkpoints	131	0.628	3	0.0242	0.813
Deadenylation-dependent mRNA decay	51	0.244	2	0.0245	0.813
Ubiquitin Mediated Degradation of Phosphorylated Cdc25A	52	0.249	2	0.0254	0.813
p53-Independent DNA Damage Response	52	0.249	2	0.0254	0.813
p53-Independent G1/S DNA damage checkpoint	52	0.249	2	0.0254	0.813
Muscle contraction	52	0.249	2	0.0254	0.813
SCF-beta-TrCP mediated degradation of Emi1	53	0.254	2	0.0263	0.813
Autodegradation of the E3 ubiquitin ligase COP1	53	0.254	2	0.0263	0.813
Stabilization of p53	54	0.259	2	0.0273	0.813
Vif-mediated degradation of APOBEC3G	55	0.264	2	0.0282	0.813
Processing of DNA ends prior to end rejoining	6	0.0288	1	0.0284	0.813
CREB phosphorylation through the activation of CaMKK	6	0.0288	1	0.0284	0.813
Host Interactions of HIV factors	141	0.676	3	0.0293	0.813
CDT1 association with the CDC6:ORC:origin complex	57	0.273	2	0.0302	0.813
SCF(Skp2)-mediated degradation of p27/p21	58	0.278	2	0.0311	0.813
Cell Cycle	508	2.43	6	0.0312	0.813
p53-Dependent G1/S DNA damage checkpoint	59	0.283	2	0.0321	0.813
p53-Dependent G1 DNA Damage Response	59	0.283	2	0.0321	0.813
Regulation of Apoptosis	59	0.283	2	0.0321	0.813
G1/S DNA Damage Checkpoints	62	0.297	2	0.0352	0.813
Assembly of the pre-replicative complex	63	0.302	2	0.0362	0.813
ER-Phagosome pathway	63	0.302	2	0.0362	0.813
Nonhomologous End-joining (NHEJ)	8	0.0383	1	0.0377	0.813
Regulation of Insulin Secretion by Free Fatty Acids	8	0.0383	1	0.0377	0.813

Regulation of Insulin Secretion by Fatty Acids Bound to GPR40 (FFAR1)	8	0.0383	1	0.0377	0.813
Multifunctional anion exchangers	8	0.0383	1	0.0377	0.813
Cyclin E associated events during G1/S transition	65	0.312	2	0.0384	0.813
Degradation of beta-catenin by the destruction complex	65	0.312	2	0.0384	0.813
Signaling by Wnt	65	0.312	2	0.0384	0.813
Activation of NF-kappaB in B Cells	66	0.316	2	0.0395	0.813
Cyclin A:Cdk2-associated events at S phase entry	66	0.316	2	0.0395	0.813
Autodegradation of Cdh1 by Cdh1:APC/C	68	0.326	2	0.0417	0.813
2-LTR circle formation	9	0.0431	1	0.0423	0.813
Axonal growth inhibition (RHOA activation)	9	0.0431	1	0.0423	0.813
eNOS activation	9	0.0431	1	0.0423	0.813
Switching of origins to a post-replicative state	69	0.331	2	0.0428	0.813
Orc1 removal from chromatin	69	0.331	2	0.0428	0.813
Cell Cycle, Mitotic	411	1.97	5	0.0439	0.813
Removal of licensing factors from origins	71	0.34	2	0.045	0.813
Regulation of DNA replication	71	0.34	2	0.045	0.813
APC/C:Cdc20 mediated degradation of Securin	71	0.34	2	0.045	0.813
Cytokine Signaling in Immune system	286	1.37	4	0.0462	0.813
Telomere Maintenance	72	0.345	2	0.0462	0.813
Methylation	10	0.0479	1	0.0469	0.813
p75NTR regulates axonogenesis	10	0.0479	1	0.0469	0.813
Signalling by NGF	290	1.39	4	0.0482	0.813
Toll Like Receptor 10 (TLR10) Cascade	74	0.355	2	0.0485	0.813
Toll Like Receptor 5 (TLR5) Cascade	74	0.355	2	0.0485	0.813
MyD88 cascade initiated on plasma membrane	74	0.355	2	0.0485	0.813

B. Enriched pathways for gradient downregulated genes

Pathway	Total	Expected	Hits	P.Value	FDR
NF-kB activation through FADD/RIP-1 pathway mediated by caspase-8 and -10	12	0.101	4	2.09E-06	0.00293
DAI mediated induction of type I IFNs	13	0.109	3	0.000151	0.0788

TRAF3-dependent IRF activation pathway	14	0.117	3	0.000191	0.0788
RIG-I/MDA5 mediated induction of IFN-alpha/beta pathways	67	0.562	5	0.000225	0.0788
TRAF6 mediated NF-kB activation	16	0.134	3	0.00029	0.0812
Cytosolic sensors of pathogen-associated DNA	19	0.159	3	0.000492	0.115
TRAF6 mediated IRF7 activation	28	0.235	3	0.00158	0.316
Negative regulators of RIG-I/MDA5 signaling	33	0.277	3	0.00255	0.447
RIP-mediated NFkB activation via DAI	11	0.0923	2	0.00362	0.564
Activation of Chaperone Genes by XBP1(S)	46	0.386	3	0.00657	0.916
Developmental Biology	417	3.5	9	0.00729	0.916
Activation of Chaperones by IRE1alpha	49	0.411	3	0.00784	0.916
Signal regulatory protein (SIRP) family interactions	20	0.168	2	0.0119	1
Signaling by FGFR1 fusion mutants	20	0.168	2	0.0119	1
cGMP effects	21	0.176	2	0.0131	1
L1CAM interactions	112	0.939	4	0.0141	1
Export of Viral Ribonucleoproteins from Nucleus	2	0.0168	1	0.0167	1
NEP/NS2 Interacts with the Cellular Export Machinery	2	0.0168	1	0.0167	1
Unfolded Protein Response	66	0.554	3	0.0176	1
IKK complex recruitment mediated by RIP1	25	0.21	2	0.0183	1
Antiviral mechanism by IFN-stimulated genes	69	0.579	3	0.0198	1
ISG15 antiviral mechanism	69	0.579	3	0.0198	1
Nitric oxide stimulates guanylate cyclase	27	0.226	2	0.0212	1
IRF3 mediated activation of type 1 IFN	3	0.0252	1	0.025	1
Beta oxidation of palmitoyl-CoA to myristoyl-CoA	3	0.0252	1	0.025	1
Cam-PDE 1 activation	3	0.0252	1	0.025	1
TGF-beta receptor signaling activates SMADs	30	0.252	2	0.0259	1
Signaling by FGFR1 mutants	31	0.26	2	0.0275	1
Cytokine Signaling in Immune system	286	2.4	6	0.0316	1
Plasmalogen biosynthesis	4	0.0335	1	0.0331	1
NGF processing	4	0.0335	1	0.0331	1
Neurophilin interactions with VEGF and VEGFR	4	0.0335	1	0.0331	1

Axon guidance	292	2.45	6	0.0345	1
TRIF-mediated TLR3/TLR4 signaling	87	0.73	3	0.0361	1
MyD88-independent cascade	88	0.738	3	0.0372	1
Toll Like Receptor 3 (TLR3) Cascade	88	0.738	3	0.0372	1
S6K1 signalling	5	0.0419	1	0.0412	1
NOSTRIN mediated eNOS trafficking	5	0.0419	1	0.0412	1
Immune System	1140	9.54	15	0.0441	1
ATM mediated phosphorylation of repair proteins	6	0.0503	1	0.0493	1
ATM mediated response to DNA double-strand break	6	0.0503	1	0.0493	1
ER Quality Control Compartment (ERQC)	6	0.0503	1	0.0493	1

C. Enriched pathways for threshold upregulated genes

Pathway	Total	Expected	Hits	P.Value	FDR
Attachment of GPI anchor to uPAR	7	0.609	4	0.0016	0.612
Activation of BAD and translocation to mitochondria	17	1.48	6	0.00227	0.612
Pre-NOTCH Transcription and Translation	12	1.04	5	0.00231	0.612
Bile salt and organic anion SLC transporters	12	1.04	5	0.00231	0.612
AKT-mediated inactivation of FOXO1A	4	0.348	3	0.00245	0.612
Transport of glucose and other sugars, bile salts and organic acids, metal ions and amine compounds	95	8.27	17	0.00307	0.612
L1CAM interactions	112	9.75	19	0.00338	0.612
AKT phosphorylates targets in the cytosol	13	1.13	5	0.00349	0.612
Regulation of Gene Expression by Hypoxia-inducible Factor	9	0.783	4	0.00501	0.731
Eicosanoids	5	0.435	3	0.00574	0.731
Acyl chain remodeling of DAG and TAG	5	0.435	3	0.00574	0.731
Proton-coupled neutral amino acid transporters	2	0.174	2	0.00756	0.731
Signal transduction by L1	35	3.05	8	0.00893	0.731
Pre-NOTCH Expression and Processing	22	1.91	6	0.00941	0.731
Transmembrane transport of small molecules	504	43.9	59	0.00997	0.731
Activation of PKB	6	0.522	3	0.0107	0.731
RSK activation	6	0.522	3	0.0107	0.731

Negative regulation of the PI3K/AKT network	11	0.957	4	0.0114	0.731
Signaling by EGFR	179	15.6	25	0.0117	0.731
Rap1 signalling	17	1.48	5	0.0125	0.731
Intrinsic Pathway for Apoptosis	37	3.22	8	0.0126	0.731
CD28 co-stimulation	30	2.61	7	0.0126	0.731
Hemostasis	511	44.5	59	0.0131	0.731
Signaling by EGFR in Cancer	181	15.7	25	0.0134	0.731
Downstream signal transduction	163	14.2	23	0.0135	0.731
Activation of BH3-only proteins	24	2.09	6	0.0146	0.731
CTLA4 inhibitory signaling	24	2.09	6	0.0146	0.731
The role of Nef in HIV-1 replication and disease pathogenesis	31	2.7	7	0.0151	0.731
Adherens junctions interactions	31	2.7	7	0.0151	0.731
Fcgamma receptor (FCGR) dependent phagocytosis	86	7.48	14	0.0157	0.733
SLC-mediated transmembrane transport	251	21.8	32	0.0173	0.74
NrCAM interactions	7	0.609	3	0.0176	0.74
Post-transcriptional Silencing By Small RNAs	7	0.609	3	0.0176	0.74
Signaling by FGFR in disease	178	15.5	24	0.0199	0.74
CD28 dependent PI3K/Akt signaling	19	1.65	5	0.0204	0.74
Transcriptional Regulation of White Adipocyte Differentiation	56	4.87	10	0.0211	0.74
Formation of apoptosome	3	0.261	2	0.0214	0.74
Downregulation of ERBB2:ERBB3 signaling	13	1.13	4	0.0215	0.74
alpha-linolenic acid (ALA) metabolism	13	1.13	4	0.0215	0.74
alpha-linolenic (omega3) and linoleic (omega6) acid metabolism	13	1.13	4	0.0215	0.74
Kinesins	41	3.57	8	0.0229	0.74
Metabolism of lipids and lipoproteins	507	44.1	57	0.024	0.74
G beta:gamma signalling through PI3Kgamma	27	2.35	6	0.0258	0.74
Lysosome Vesicle Biogenesis	27	2.35	6	0.0258	0.74
Lysine catabolism	8	0.696	3	0.0263	0.74
Vitamin C (ascorbate) metabolism	8	0.696	3	0.0263	0.74
Nicotinate metabolism	8	0.696	3	0.0263	0.74

Linoleic acid (LA) metabolism	8	0.696	3	0.0263	0.74
Signaling by ERBB2	164	14.3	22	0.0264	0.74
DAP12 signaling	164	14.3	22	0.0264	0.74
Regulation of Lipid Metabolism by Peroxisome proliferator-activated receptor alpha (PPARalpha)	76	6.61	12	0.0301	0.826
HIV Infection	214	18.6	27	0.0308	0.826
Cell-cell junction organization	60	5.22	10	0.0327	0.826
Downstream signaling of activated FGFR	150	13.1	20	0.0351	0.826
NGF signalling via TRKA from the plasma membrane	207	18	26	0.0352	0.826
Synthesis of Prostaglandins (PG) and Thromboxanes (TX)	15	1.31	4	0.0357	0.826
Signalling to p38 via RIT and RIN	15	1.31	4	0.0357	0.826
Developmental Biology	417	36.3	47	0.0369	0.826
AKT phosphorylates targets in the nucleus	9	0.783	3	0.037	0.826
Signaling by PDGF	189	16.4	24	0.0376	0.826
Signalling to ERKs	37	3.22	7	0.0377	0.826
ABC-family proteins mediated transport	37	3.22	7	0.0377	0.826
Arachidonic acid metabolism	45	3.92	8	0.0381	0.826
Signaling by SCF-KIT	142	12.4	19	0.0381	0.826
Proton-coupled monocarboxylate transport	4	0.348	2	0.0403	0.826
Signaling by FGFR	162	14.1	21	0.041	0.826
G-protein beta:gamma signalling	30	2.61	6	0.0414	0.826
Platelet activation, signaling and aggregation	220	19.1	27	0.0416	0.826
Cell junction organization	89	7.74	13	0.0432	0.826
ARMS-mediated activation	16	1.39	4	0.0444	0.826
Late Phase of HIV Life Cycle	108	9.4	15	0.0463	0.826
Transcription-coupled NER (TC-NER)	47	4.09	8	0.0477	0.826
Dual incision reaction in TC-NER	31	2.7	6	0.0478	0.826
Formation of transcription-coupled NER (TC-NER) repair complex	31	2.7	6	0.0478	0.826
Amino acid transport across the plasma membrane	31	2.7	6	0.0478	0.826
Trafficking of AMPA receptors	31	2.7	6	0.0478	0.826
Glutamate Binding, Activation of AMPA Receptors	31	2.7	6	0.0478	0.826

and Synaptic Plasticity					
Adaptive Immune System	654	56.9	69	0.0478	0.826
trans-Golgi Network Vesicle Budding	64	5.57	10	0.0483	0.826
Clathrin derived vesicle budding	64	5.57	10	0.0483	0.826
MHC class II antigen presentation	118	10.3	16	0.0488	0.826
RAF/MAP kinase cascade	10	0.87	3	0.0495	0.826
Zinc influx into cells by the SLC39 gene family	10	0.87	3	0.0495	0.826
GP1b-IX-V activation signalling	10	0.87	3	0.0495	0.826

D. Enriched pathways for threshold downregulated genes

Pathway	Total	Expected	Hits	P.Value	FDR
Cell Cycle	508	49.4	71	0.000831	0.35
Class I MHC mediated antigen processing & presentation	267	26	42	0.00105	0.35
Gene Expression	1090	106	134	0.00115	0.35
Nuclear Envelope Reassembly	14	1.36	6	0.00125	0.35
Initiation of Nuclear Envelope Reformation	14	1.36	6	0.00125	0.35
Cell Cycle Checkpoints	131	12.7	24	0.00161	0.377
Platelet activation, signaling and aggregation	220	21.4	35	0.0022	0.441
Immune System	1140	111	136	0.00371	0.563
Metabolism of nucleotides	81	7.87	16	0.00433	0.563
Cell Cycle, Mitotic	411	39.9	56	0.00512	0.563
Phospholipid metabolism	135	13.1	23	0.00517	0.563
N-glycan trimming in the ER and Calnexin/Calreticulin cycle	13	1.26	5	0.00566	0.563
G1/S DNA Damage Checkpoints	62	6.03	13	0.00576	0.563
Pyrimidine metabolism	24	2.33	7	0.00629	0.563
Rho GTPase cycle	123	12	21	0.00718	0.563
Signaling by Rho GTPases	123	12	21	0.00718	0.563
Metabolism of RNA	339	33	47	0.00724	0.563
Glycerophospholipid biosynthesis	86	8.36	16	0.0079	0.563
Antigen Presentation: Folding, assembly and peptide loading of class I MHC	25	2.43	7	0.00802	0.563

Response to elevated platelet cytosolic Ca2+	94	9.14	17	0.00838	0.563
Deadenylation-dependent mRNA decay	51	4.96	11	0.00861	0.563
Antigen processing: Ubiquitination & Proteasome degradation	224	21.8	33	0.00957	0.563
Signaling by FGFR1 fusion mutants	20	1.94	6	0.00972	0.563
p53-Dependent G1/S DNA damage checkpoint	59	5.73	12	0.01	0.563
p53-Dependent G1 DNA Damage Response	59	5.73	12	0.01	0.563
Platelet degranulation	89	8.65	16	0.011	0.592
Clearance of Nuclear Envelope Membranes from Chromatin	10	0.972	4	0.0115	0.598
Transcription-coupled NER (TC-NER)	47	4.57	10	0.0132	0.635
Metabolism of mRNA	317	30.8	43	0.0144	0.635
Toll-Like Receptors Cascades	123	12	20	0.0145	0.635
Mitotic M-M/G1 phases	266	25.9	37	0.0154	0.635
Mitotic Anaphase	198	19.2	29	0.0157	0.635
M Phase	233	22.6	33	0.0167	0.635
ER-Phagosome pathway	63	6.12	12	0.0167	0.635
Vitamin B5 (pantothenate) metabolism	11	1.07	4	0.0167	0.635
Calnexin/calreticulin cycle	11	1.07	4	0.0167	0.635
Mitotic Metaphase and Anaphase	199	19.3	29	0.0168	0.635
Nuclear Envelope Breakdown	17	1.65	5	0.0196	0.703
G alpha (12/13) signalling events	80	7.78	14	0.0209	0.703
Regulation of mRNA Stability by Proteins that Bind AU-rich Elements	88	8.55	15	0.0214	0.703
G1/S Transition	113	11	18	0.0239	0.703
Deadenylation of mRNA	24	2.33	6	0.0242	0.703
Signaling by NOTCH1 t(7;9)(NOTCH1:M1580_K2555) Translocation Mutant	74	7.19	13	0.0247	0.703
Signaling by NOTCH1 in Cancer	74	7.19	13	0.0247	0.703
Signaling by NOTCH1 PEST Domain Mutants in Cancer	74	7.19	13	0.0247	0.703
FBXW7 Mutants and NOTCH1 in Cancer	74	7.19	13	0.0247	0.703
Signaling by NOTCH1 HD Domain Mutants in	74	7.19	13	0.0247	0.703

Cancer					
Signaling by NOTCH1 HD+PEST Domain Mutants in Cancer	74	7.19	13	0.0247	0.703
Signaling by NOTCH1	74	7.19	13	0.0247	0.703
Synthesis of PC	18	1.75	5	0.0251	0.703
Formation of the active cofactor, UDP-glucuronate	3	0.292	2	0.0265	0.728
Mitotic G1-G1/S phases	140	13.6	21	0.0287	0.729
Autodegradation of Cdh1 by Cdh1:APC/C	68	6.61	12	0.0293	0.729
Nucleotide Excision Repair	53	5.15	10	0.0294	0.729
APC/C:Cdc20 mediated degradation of mitotic proteins	76	7.39	13	0.0301	0.729
Host Interactions of HIV factors	141	13.7	21	0.0308	0.729
Activated TLR4 signalling	100	9.72	16	0.0309	0.729
Sphingolipid de novo biosynthesis	32	3.11	7	0.031	0.729
Caspase-mediated cleavage of cytoskeletal proteins	13	1.26	4	0.031	0.729
Triglyceride Biosynthesis	39	3.79	8	0.0312	0.729
Stabilization of p53	54	5.25	10	0.033	0.743
Activation of APC/C and APC/C:Cdc20 mediated degradation of mitotic proteins	77	7.48	13	0.0332	0.743
Signalling by NGF	290	28.2	38	0.0334	0.743
Coenzyme A biosynthesis	8	0.778	3	0.0353	0.762
Signaling by TGF-beta Receptor Complex	70	6.8	12	0.0358	0.762
GPVI-mediated activation cascade	33	3.21	7	0.0361	0.762
Antigen processing-Cross presentation	78	7.58	13	0.0364	0.762
Adaptive Immune System	654	63.6	77	0.0386	0.771
Toll Like Receptor 4 (TLR4) Cascade	103	10	16	0.0393	0.771
APC/C:Cdc20 mediated degradation of Securin	71	6.9	12	0.0395	0.771
Synthesis of DNA	95	9.23	15	0.0397	0.771
Cytokine Signaling in Immune system	286	27.8	37	0.0419	0.771
S Phase	122	11.9	18	0.0469	0.771
Cyclin E associated events during G1/S transition	65	6.32	11	0.047	0.771
Loss of proteins required for interphase microtubule organizationÃ‚Â from the centrosome	65	6.32	11	0.047	0.771

Loss of Nlp from mitotic centrosomes	65	6.32	11	0.047	0.771
MyD88:Mal cascade initiated on plasma membrane	81	7.87	13	0.0475	0.771
Toll Like Receptor TLR1:TLR2 Cascade	81	7.87	13	0.0475	0.771
Toll Like Receptor TLR6:TLR2 Cascade	81	7.87	13	0.0475	0.771
Toll Like Receptor 2 (TLR2) Cascade	81	7.87	13	0.0475	0.771
Hemostasis	511	49.7	61	0.049	0.771
Pentose phosphate pathway (hexose monophosphate shunt)	9	0.875	3	0.0492	0.771
Pyrimidine salvage reactions	9	0.875	3	0.0492	0.771
Regulation of cytoskeletal remodeling and cell spreading by IPP complex components	9	0.875	3	0.0492	0.771
NOTCH1 Intracellular Domain Regulates Transcription	50	4.86	9	0.0492	0.771
Assembly of the RAD50-MRE11-NBS1 complex at DNA double-strand breaks	4	0.389	2	0.0496	0.771
MRN complex relocalizes to nuclear foci	4	0.389	2	0.0496	0.771
Alpha-oxidation of phytanate	4	0.389	2	0.0496	0.771

Supplemental codes for reanalysis of single cell RNA sequencing data on R:

R code for analysis of GSE163668

```
> library(Seurat)

> library(Matrix)

> library(dplyr)

>data_dir<- 'C:/Users/dclabiicb/Documents/GSE163668 pooled 5,6'

>expression_matrix<- Read10X(data.dir = data_dir)

>sample.tmp.seurat<- CreateSeuratObject(counts = expression_matrix, min.cells = 3, min.features =
200)

>sample.tmp.seurat<- NormalizeData(sample.tmp.seurat)

>sample.tmp.seurat<- FindVariableFeatures(sample.tmp.seurat, selection.method = "vst", nfeatures
= 4000)

>nCoV.integrated<- ScaleData(sample.tmp.seurat)

>nCoV.integrated<- RunPCA(nCoV.integrated, verbose = FALSE,npcs = 100)

>nCoV.integrated<- ProjectDim(object = nCoV.integrated)

>nCoV.integrated<- FindNeighbors(object = nCoV.integrated, dims = 1:50)

>nCoV.integrated<- FindClusters(object = nCoV.integrated, resolution = 1.2)

>nCoV.integrated<- RunTSNE(object = nCoV.integrated, dims = 1:50)

>DimPlot(object = nCoV.integrated, reduction = 'tsne',label = TRUE)

>print(FeaturePlot(object = nCoV.integrated, features = c('PLAUR', 'ITGAX', 'HLA-DRA'),cols =
c("lightgrey","Blue")))

>HLADRA_expression = GetAssayData(object = nCoV.integrated)["HLA-DRA",]

>ITGAX_expression = GetAssayData(object = nCoV.integrated)["ITGAX",]

>neg_ids = names(which(HLADRA_expression<0.5))

>neg_cells = subset(nCoV.integrated,cells=neg_ids)

>pos_ids = names(which(ITGAX_expression>0.5))

>pos_cells = subset(neg_cells,cells=pos_ids)

>FeaturePlot(pos_cells,"PLAUR", cols=c("lightgrey", "red"))

>neg_ids = names(which(HLADRA_expression<1))

>neg_cells = subset(nCoV.integrated,cells=neg_ids)

>pos_ids = names(which(ITGAX_expression>0.3))

>pos_cells = subset(neg_cells,cells=pos_ids)
```

```
>FeaturePlot(pos_cells,"PLAUR", cols=c("lightgrey", "red"))
```

R code for analysis of GSE145926

```
> library(Seurat)

> library(Matrix)

> library(dplyr)

> expression_matrix1 <- Read10X_h5(choose.files())

>sample.tmp.seurat1 <- CreateSeuratObject(counts = expression_matrix1, min.cells = 3,
min.features = 200)

> expression_matrix2 <- Read10X_h5(choose.files())

>sample.tmp.seurat2 <- CreateSeuratObject(counts = expression_matrix2, min.cells = 3,
min.features = 200)

>sample.tmp.seurat.combined<- merge(sample.tmp.seurat1, y = sample.tmp.seurat2)

> expression_matrix3 <- Read10X_h5(choose.files())

>sample.tmp.seurat3 <- CreateSeuratObject(counts = expression_matrix3, min.cells = 3,
min.features = 200)

>sample.tmp.seurat.combined.mild<- merge(sample.tmp.seurat.combined, y = sample.tmp.seurat3)

>sample.tmp.seurat.combined.mild<- NormalizeData(sample.tmp.seurat.combined.mild, verbose =
FALSE)

>sample.tmp.seurat.combined.mild<- FindVariableFeatures(sample.tmp.seurat.combined.mild,
selection.method = "vst", nfeatures = 2000,verbose = FALSE)

>nCoV.integrated<- ScaleData(sample.tmp.seurat.combined.mild, verbose = FALSE)

>nCoV.integrated<- RunPCA(nCoV.integrated, verbose = FALSE,npcs = 100)

>nCoV.integrated.mild<- ProjectDim(object = nCoV.integrated)

>nCoV.integrated<- FindNeighbors(object = nCoV.integrated, dims = 1:50)

>nCoV.integrated<- FindClusters(object = nCoV.integrated, resolution = 0.5)

>nCoV.integrated<- RunTSNE(object = nCoV.integrated, dims = 1:50)

>DimPlot(object = nCoV.integrated, reduction = 'tsne',label = TRUE)

>FeaturePlot(nCoV.integrated,features=c("ITGAX", "HLA-DRA", "PLAUR"), cols=c("light Grey",
"Blue"))

>VlnPlot(object = nCoV.integrated, features = c("ITGAX"),pt.size = 0)

>VlnPlot(object = nCoV.integrated, features = c("HLA-DRA"),pt.size = 0)

>neg_cells = subset(nCoV.integrated,cells=neg_ids)

>pos_ids = names(which(ITGAX_expression>0.5))
```

```
>pos_cells = subset(neg_cells,cells=pos_ids)
>FeaturePlot(pos_cells,"PLAUR", cols=c("light Grey", "Red"))
```

R code for analysis of GSE168710

```
>Raw.data<- readRDS(choose.files())
> metadata <- readRDS(choose.files())
> data <- CreateSeuratObject(Raw.data, meta.data = metadata, min.cells = 3, min.features = 200)
> data <- FindVariableFeatures(data, selection.method = "vst", nfeatures = 4000, verbose = FALSE)
> data <- ScaleData(data, verbose = FALSE)
>nCoV.integrated<- RunPCA(data, verbose = FALSE,npcs = 100)
>nCoV.integrated<- FindNeighbors(object = nCoV.integrated, dims = 1:50)
>nCoV.integrated<- FindClusters(object = nCoV.integrated, resolution = 1.2)
>nCoV.integrated<- RunTSNE(object = nCoV.integrated, dims = 1:50)
>DimPlot(object = nCoV.integrated, reduction = 'tsne',label = TRUE)
>DimPlot(object = nCoV.integrated, reduction = 'tsne',group.by= "stim",label = TRUE)
>FeaturePlot(nCoV.integrated,features=c("PLAUR"), cols=c("light Grey", "Blue"),label=TRUE)
>VlnPlot(object = nCoV.integrated, features = c("HLA-DRA"),pt.size = 0)
>VlnPlot(object = nCoV.integrated, features = c("ITGAX"),pt.size = 0)
>HLADRA_expression = GetAssayData(object = nCoV.integrated)["HLA-DRA",]
>ITGAX_expression = GetAssayData(object = nCoV.integrated)["ITGAX",]
>neg_ids = names(which(HLADRA_expression<2.5))
>neg_cells = subset(nCoV.integrated,cells=neg_ids)
>pos_ids = names(which(ITGAX_expression>0.8))
>pos_cells = subset(neg_cells,cells=pos_ids)
>FeaturePlot(pos_cells,features=c("PLAUR"), cols=c("light Grey", "Blue"))
>VlnPlot(pos_cells, features = c("PLAUR"),pt.size = 0)
>PLAUR_expression = GetAssayData(object = nCoV.integrated)["PLAUR",]
> PLAUR_2_ids = names(which(PLAUR_expression>2.2))
>  PLAUR_2_cells = subset(nCoV.integrated,cells=PLAUR_2_ids)
>DimPlot(object = PLAUR_2_cells, reduction = 'tsne',group.by= "stim",label = TRUE)
```

Supplemental references:

39. Shaath H et al. Single-Cell Transcriptome Analysis Highlights a Role for Neutrophils and Inflammatory Macrophages in the Pathogenesis of Severe COVID-19. Cells. 2020 Oct 29;9(11):2374. doi: 10.3390/cells9112374.

40. O'Brien KD et al. Glycosylphosphatidylinositol-specific phospholipase D is expressed by macrophages in human atherosclerosis and colocalizes with oxidation epitopes. Circulation. 1999 Jun 8;99(22):2876-82. doi: 10.1161/01.cir.99.22.2876.

41. Etscheid M et al. Altered structure and function of fibrinogen after cleavage by Factor VII Activating Protease (FSAP). Biochim Biophys Acta Mol Basis Dis. 2018 Oct;1864(10):3397-3406. doi: 10.1016/j.bbadis.2018.07.030.

42. Subramaniam S et al. Defective thrombus formation in mice lacking endogenous factor VII activating protease (FSAP). Thromb Haemost. 2015 Apr;113(4):870-80. doi: 10.1160/TH14-06-0519.

43. de Jong HK et al. Diabetes-independent increase of factor VII-activating protease activation in patients with Gram-negative sepsis (melioidosis). J Thromb Haemost. 2015 Jan;13(1):41-6. doi: 10.1111/jth.12776.

44. Ma Y et al. ITIH4, as an inflammation biomarker, mainly increases in bacterial bloodstream . Cytokine. 2021 Feb;138:155377. doi: 10.1016/j.cyto.2020.155377.

45. Lee K-Y et al. Inter-alpha-trypsin inhibitor heavy chain 4: a novel biomarker for environmental exposure to particulate air pollution in patients with chronic obstructive pulmonary disease. Int J Chron Obstruct Pulmon Dis. 2015 Apr 24;10:831-41. doi: 10.2147/COPD.S81611.

46. Pihl R et al. ITIH4 acts as a protease inhibitor by a novel inhibitory mechanism. Sci Adv. 2021 Jan 8;7(2):eaba7381. doi: 10.1126/sciadv.aba7381.

47. Baralla A et al. Plasma Proteomic Signatures in Early Chronic Obstructive Pulmonary Disease. Proteomics Clin Appl. 2018 May;12(3):e1700088. doi: 10.1002/prca.201700088.

48. Wajda A et al. Tissue factor and human apolipoprotein H genetic variants and pro-inflammatory cytokines in systemic lupus erythematosus patients. Clin Exp Rheumatol. May-Jun 2021;39(3):587-600.

49. Navab M, Anantharamaiah GM, Fogelman AM. The role of high-density lipoprotein in inflammation. Trends Cardiovasc Med. 2005 May;15(4):158-61. doi: 10.1016/j.tcm.2005.05.008.

50. Fuior EV, Gafencu AV. Apolipoprotein C1: Its Pleiotropic Effects in Lipid Metabolism and Beyond. Int J Mol Sci. 2019 Nov 26;20(23):5939. doi: 10.3390/ijms20235939.

51. Berbée JFP et al. Apolipoprotein CI stimulates the response to lipopolysaccharide and reduces mortality in gram-negative sepsis. FASEB J. 2006 Oct;20(12):2162-4. doi: 10.1096/fj.05-5639fje.

52. Routhier EL et al. Human BIN3 complements the F-actin localization defects caused by loss of Hob3p, the fission yeast homolog of Rvs161p. J Biol Chem. 2001 Jun 15;276(24):21670-7. doi: 10.1074/jbc.M101096200.

53. Hudalla H et al. Carbonic Anhydrase Inhibition Ameliorates Inflammation and Experimental Pulmonary Hypertension. Am J Respir Cell Mol Biol. 2019 Oct;61(4):512-524. doi: 10.1165/rcmb.2018-0232OC.

54. Henry EK et al. Carbonic anhydrase enzymes regulate mast cell-mediated inflammation. J Exp Med. 2016 Aug 22;213(9):1663-73. doi: 10.1084/jem.20151739.

55. Farjo KM et al. Retinol-binding protein 4 induces inflammation in human endothelial cells by an NADPH oxidase- and nuclear factor kappa B-dependent and retinol-independent mechanism. Mol Cell Biol. 2012 Dec;32(24):5103-15. doi: 10.1128/MCB.00820-12

56. Scavenius C et al. The Compact and Biologically Relevant Structure of Inter-α-inhibitor Is Maintained by the Chondroitin Sulfate Chain and Divalent Cations. J Biol Chem. 2016 Feb 26;291(9):4658-70. doi: 10.1074/jbc.M115.678748.

57. Lord MS et al. The Inter-α-Trypsin Inhibitor Family: Versatile Molecules in Biology and Pathology. J Histochem Cytochem. 2020 Dec;68(12):907-927. doi: 10.1369/0022155420940067.

58. Fang S et al. Decreased complement C3 levels are associated with poor prognosis in patients with COVID-19: A retrospective cohort study. Int Immunopharmacol. 2020 Dec;89(Pt A):107070. doi: 10.1016/j.intimp.2020.107070.

59. Spicer BA et al. The first transmembrane region of complement component-9 acts as a brake on its self-assembly. Nat Commun. 2018 Aug 15;9(1):3266. doi: 10.1038/s41467-018-05717-0.

60. Soames CJ, Sim RB. Interactions between human complement components factor H, factor I and C3b. Biochem J. 1997 Sep 1;326 (Pt 2)(Pt 2):553-61. doi: 10.1042/bj3260553.

61. Lachmann PJ, Müller-Eberhard HJ. The demonstration in human serum of "conglutinogen-activating factor" and its effect on the third component of complement. J Immunol. 1968 Apr;100(4):691-8.

62. Kavanagh D, Goodship TH, Richards A. Atypical hemolytic uremic syndrome. Semin Nephrol. 2013 Nov;33(6):508-30. doi: 10.1016/j.semnephrol.2013.08.003.

63. Morena-Barrio ME et al. Prognostic value of thrombin generation parameters in hospitalized COVID-19 patients. Sci Rep. 2021 Apr 8;11(1):7792. doi: 10.1038/s41598-021-85906-y.

64. Zhou A et al. How vitronectin binds PAI-1 to modulate fibrinolysis and cell migration. Nat Struct Biol. 2003 Jul;10(7):541-4. doi: 10.1038/nsb943.

65. Conti F et al. Expression of the membrane attack complex of complement and its inhibitors during human liver allograft transplantation. J Hepatol. 1997 Nov;27(5):881-9. doi: 10.1016/s0168-8278(97)80326-1.

66. Watanabe H et al. An acute phase protein α 1-acid glycoprotein mitigates AKI and its progression to CKD through its anti-inflammatory action. Sci Rep. 2021 Apr 12;11(1):7953. doi: 10.1038/s41598-021-87217-8.

67. Ceciliani F, Lecchi C.The Immune Functions of α 1 Acid Glycoprotein. Curr Protein Pept Sci. 2019;20(6):505-524. doi: 10.2174/1389203720666190405101138.

68. Zhang Y et al. Coagulopathy and Antiphospholipid Antibodies in Patients with Covid-19. N Engl J Med. 2020 Apr 23;382(17):e38. doi: 10.1056/NEJMc2007575.

69. Yin ET, Wessler S, Stoll PJ. Identity of plasma-activated factor X inhibitor with antithrombin 3 and heparin cofactor. J Biol Chem. 1971 Jun 10;246(11):3712-9.

70. Matsumoto H et al. Decreased antithrombin activity in the early phase of trauma is strongly associated with extravascular leakage, but not with antithrombin consumption: a prospective observational study. Thromb J. 2018 Aug 1;16:17. doi: 10.1186/s12959-018-0171-7.

71. Pilling D, Gomer RH. The Development of Serum Amyloid P as a Possible Therapeutic. Front Immunol. 2018 Oct 16;9:2328. doi: 10.3389/fimmu.2018.02328.
